# *HACD1*, a regulator of membrane composition and fluidity, promotes myoblast fusion and skeletal muscle growth

**DOI:** 10.1093/jmcb/mjv049

**Published:** 2015-07-09

**Authors:** Jordan Blondelle, Yusuke Ohno, Vincent Gache, Stéphane Guyot, Sébastien Storck, Nicolas Blanchard-Gutton, Inès Barthélémy, Gemma Walmsley, Anaëlle Rahier, Stéphanie Gadin, Marie Maurer, Laurent Guillaud, Alexandre Prola, Arnaud Ferry, Geneviève Aubin-Houzelstein, Jean Demarquoy, Frédéric Relaix, Richard J. Piercy, Stéphane Blot, Akio Kihara, Laurent Tiret, Fanny Pilot-Storck

**Affiliations:** 1 Inserm, IMRB U955-E10, 94000 Créteil, France; 2 Université Paris-Est, Ecole nationale vétérinaire d'Alfort (EnvA), 94700 Maisons-Alfort, France; 3 Université Paris-Est Créteil, Faculté de médecine, 94000 Créteil, France; 4 Laboratory of Biochemistry, Faculty of Pharmaceutical Sciences, Hokkaido University, Sapporo 060-0812, Japan; 5 Université de Bourgogne, UMR A 02.102 PAM-EPMB, AgroSup Dijon, 21000 Dijon, France; 6 Institut Necker-Enfants Malades, INSERM U1151-CNRS UMR 8253, Sorbonne Paris Cité, Université Paris Descartes, Faculté de Médecine-Site Broussais, 75015 Paris, France; 7 Comparative Neuromuscular Disease Laboratory, Department of Clinical Sciences and Services, Royal Veterinary College, London NW1 0TU, UK; 8 Thérapie des maladies du muscle strié INSERM U974 - CNRS UMR7215 - UPMC UM76 - Institut de Myologie, Université Pierre et Marie Curie - Université Paris Descartes, 75000 Paris, France; 9 Université de Bourgogne, Faculté des Sciences Gabriel, Bio-PeroxIL, 21000 Dijon, France

**Keywords:** centronuclear myopathy, LPC, MUFA, PTPLA, VLCFA

## Abstract

The reduced diameter of skeletal myofibres is a hallmark of several congenital myopathies, yet the underlying cellular and molecular mechanisms remain elusive. In this study, we investigate the role of *HACD1/PTPLA*, which is involved in the elongation of the very long chain fatty acids, in muscle fibre formation. In humans and dogs, HACD1 deficiency leads to a congenital myopathy with fibre size disproportion associated with a generalized muscle weakness. Through analysis of HACD1-deficient Labradors, *Hacd1*-knockout mice, and *Hacd1*-deficient myoblasts, we provide evidence that HACD1 promotes myoblast fusion during muscle development and regeneration. We further demonstrate that in normal differentiating myoblasts, expression of the catalytically active HACD1 isoform, which is encoded by a muscle-enriched splice variant, yields decreased lysophosphatidylcholine content, a potent inhibitor of myoblast fusion, and increased concentrations of ≥C18 and monounsaturated fatty acids of phospholipids. These lipid modifications correlate with a reduction in plasma membrane rigidity. In conclusion, we propose that fusion impairment constitutes a novel, non-exclusive pathological mechanism operating in congenital myopathies and reveal that *HACD1* is a key regulator of a lipid-dependent muscle fibre growth mechanism.

## Introduction

Congenital myopathies are rare hereditary diseases often characterized by muscle weakness leading to physical motor impairment that ranges from mild to life-threatening disabilities (North, 2008). Several of these myopathies, including myotubular/centronuclear myopathies (CNM) and other myopathies with congenital fibre size disproportion, demonstrate early myofibre hypotrophy (Jungbluth et al., 2008; North, 2008; Romero, 2010). However, the underlying pathogenic mechanisms remain largely unknown.

In dogs and humans, *3-hydroxyacyl-CoA dehydratase 1* (*HACD1*) deficiency has been associated with recessive congenital myopathies characterized by early-onset muscle weakness (Pelé et al., 2005; Muhammad et al., 2013). Early postnatal heterogeneity in myofibre size has been reported in both species, whereas progressive nuclear centralization has been observed in diseased dogs, a hallmark of CNM (Tiret et al., 2003). *HACD1* encodes an endoplasmic reticulum (ER)-resident enzyme that interacts with ELOVL1-7, KAR and TER proteins to form a complex involved in the synthesis of very long chain fatty acids (VLCFAs) (Ikeda et al., 2008; Ohno et al., 2010). As opposed to fatty acids up to C16, synthesized by a cytosolic fatty acid synthase, the longer VLCFAs are elongated within the ER in a four-step cycle (Kihara, 2012). In mammals, four paralogous genes encoding HACD1-4 catalyse the third step of this elongation cycle (Ikeda et al., 2008).

Following their incorporation into membrane lipids (such as phospholipids and sphingolipids), VLCFAs elicit specific functions based on their chain length and degree of unsaturation (Guillou et al., 2010; Kihara, 2012). In particular, they promote strong membrane curvature and vesicle fusion (Schneiter et al., 2004; Molino et al., 2014). In yeast, VLCFA elongation complex displays physical interactions with the desaturase enzyme that catalyses the unsaturation of fatty acids (Miller et al., 2005), indicating interconnections between pathways involved in lipid balance. Although *Hacd1* deficiency was reported to impair myoblast growth and differentiation *in vitro* (Lin et al., 2012), no molecular role for HACD1 has yet been reported in muscle cells.

We hypothesized that heterogeneity of muscle fibre size in *HACD1*-related myopathies reflects a defect in muscle fibre development, due to altered lipid balance. Muscle fibre development occurs from embryogenesis through the early postnatal development and can be recapitulated in adults following muscle injury. During myofibre development, mononucleated precursor cells, called myoblasts, differentiate through a well-defined process (Buckingham, 2001; Bentzinger et al., 2012). Myoblasts are derived from embryonic precursors or adult muscle satellite stem cells and once activated, are committed to a myogenic programme via a cascade of transcription factor activation that triggers myoblast fusion, leading to the formation of large, multinucleated myofibres able to generate contractile force. A wealth of accumulated *in vivo* data strongly suggests that myoblast fusion is a limiting step for optimal myofibre growth, muscle mass, and regenerative capacity (Horsley et al., 2001; Doherty et al., 2005; Georgiadis et al., 2007; Laurin et al., 2008; Hochreiter-Hufford et al., 2013; Millay et al., 2013, 2014; Lenhart et al., 2014). Similar to other processes involving membrane fusion (Chernomordik and Kozlov, 2005), myoblast fusion is the result of a complex interplay between lipids and proteins, which specific roles are just now being unravelled (Abmayr and Pavlath, 2012; Aguilar et al., 2013). A drop in membrane rigidity, known to promote membrane fusion in other cellular contexts (Maxfield and Tabas, 2005), is observed just before myoblast fusion (Prives and Shinitzky, 1977), and modifications in membrane lipid composition, putatively yielding a raise in membrane fluidity, have been reported during myoblast differentiation (Nakanishi et al., 2001; Briolay et al., 2013). Giving further support that lipids have an important role in regulating membrane fusion, adding unsaturated fatty acids to the culture media that purportedly increase membrane fluidity increases the capacity of myoblasts to fuse (Prives and Shinitzky, 1977; Nakanishi et al., 2001; Lee et al., 2009; Briolay et al., 2013). In contrast, lysophosphatidylcholine, which inhibits lipid bilayer fusion due to its inverted cone shape (Chernomordik and Kozlov, 2005), prevents myoblast fusion when added to culture media (Leikina et al., 2013). Altogether, these data suggest a model where naturally occurring modifications of the lipid membrane composition and concomitant alterations in fluidity would play key role in myoblast fusion. However, little is known regarding the genetic control of these modifications; in addition, the physiological and clinical relevance of modifications in composition and fluidity of myoblast membrane has yet to be investigated.

Given the enzymatic function of HACD1 and its implication in muscle homeostasis, we decided to address its precise role in muscle fibre development in the light of its molecular properties. We investigated this role by using combined *in vitro* and *in vivo* experiments in mice and dogs.

## Results

### HACD1 is required for muscle fibre growth

**Figure 1:**
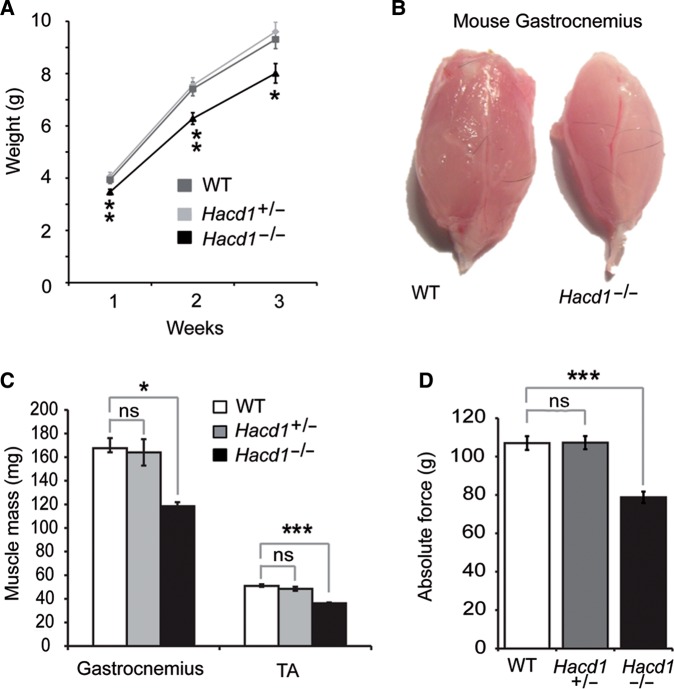
Myopathy in *Hacd1^−/−^* mice. (**A**) Weight gain in *Hacd1^−/−^*, *Hacd1^+/−^*, and wild-type (WT) mice from birth to postnatal week 3 (*n* = 11, 15, and 12, respectively). (**B**) Photograph of *gastrocnemius* muscles from WT (left) and *Hacd1^−/−^* (right) 6-month-old mice. (**C**) Mean mass of *gastrocnemius* and *tibialis anterior* (TA) muscles from 6-month-old *Hacd1^−/−^*, *Hacd1^+/−^*, and WT mice (*gastrocnemius*: *n* = 5, 3, and 4 mice, respectively; TA: *n* = 9, 7, and 8 mice, respectively). (**D**) Mean absolute maximal force of TA muscles from *Hacd1^−/−^*, *Hacd1^+/−^*, and WT mice (*n* = 16, 12, and 16, respectively). Error bars correspond to standard error of the mean. *P < 0.05, **P < 0.01, ***P < 0.001.

*HACD1* loss-of-function mutations have been associated with congenital myopathies in humans and dogs (Pelé et al., 2005; Muhammad et al., 2013). To confirm the causative role of HACD1 deficiency in skeletal muscle impairment, we generated *Hacd1*-knockout mice using a recombinant null allele inserting the *LacZ* reporter gene (Supplementary Figure S1A–C). Heterozygous mice for the knockout allele were morphologically and histologically indistinguishable from their wild-type littermates, in accordance with the recessive inheritance pattern of *HACD1* mutation in human and dog (Pelé et al., 2005; Muhammad et al., 2013); they were used alongside wild-type mice as controls. In contrast, homozygous mice for the knockout allele (hereafter *Hacd1^−/−^* mice), although viable and fertile, presented a reduced body weight gain after birth (Figure 1A). At 1 week of age, body weight of *Hacd1^−/−^* mice was reduced by 14.6% compared with control mice (*P* < 0.01). The reduced weight gain was never compensated in adulthood despite a normal body size (Supplementary Figure S1D), with a 15.2% and a 16.4% reduction in body weight at 12 weeks and 6 months of age compared with control mice, respectively (*P* < 0.05 and *P* < 0.01, respectively). This reduction in body weight could be more specifically attributed to a 29% reduction in muscle weight (Figure 1B and C) and correlated with a 27.5% reduction in the muscle absolute maximal force compared with controls (Figure 1D), however leaving specific maximal force unchanged (Supplementary Figure S1E). In accordance with a deleterious muscle weakness, *Hacd1^−/−^* mice often displayed kyphosis (Supplementary Figure S1F).

**Figure 2:**
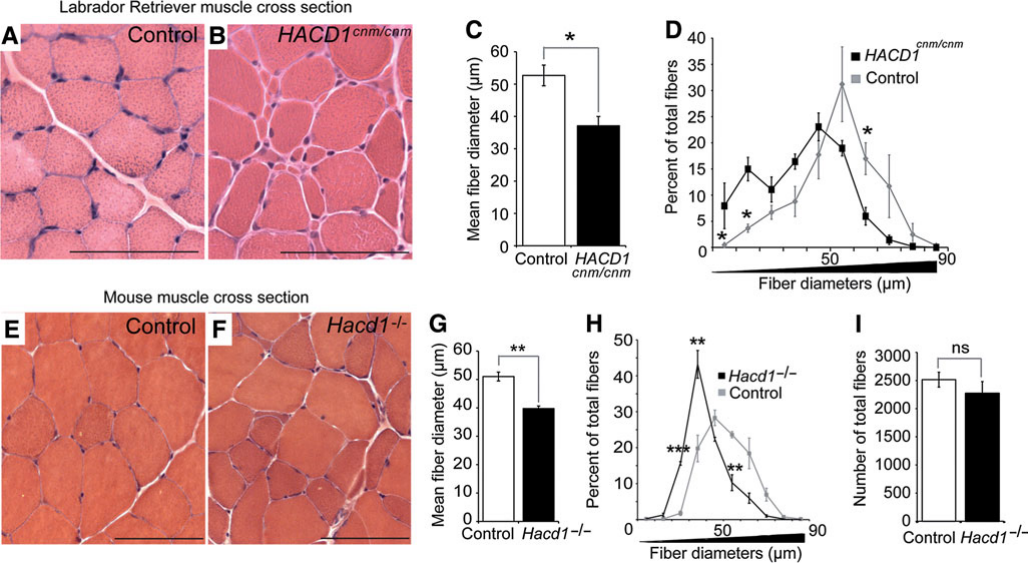
Defective myofibre growth in HACD1-deficient dogs and mice. (**A** and **B**) H&E-stained transverse sections of *biceps femoris* muscles from 5-month-old control (**A**) and *HACD1^cnm/cnm^* CNM-affected (**B**) dogs showing hypotrophic fibres in **B**. (**C**) Average myofibre diameter (in μm) in 3- to 4-year-old control and *HACD1^cnm/cnm^* dogs (*n* = 3 for each genotype; ≥460 fibres examined per dog). (**D**) Percent distribution by diameter (μm) of myofibres analysed in **C**. (**E** and **F**) H&E-stained transverse sections of TA muscles from 3-month-old control (i.e. WT or *Hacd1^+/−^* in **E**) and *Hacd1^−/−^* (**F**) mice showing fibre size disproportion in **F** (*n* = 3 for each condition). (**G**) Mean fibre diameter in TA muscles from 3-month-old control and *Hacd1^−/−^* mice (*n* = 3 for each group; ≥780 fibres per mouse). (**H**) Percent distribution by diameter (μm) of myofibres analysed in **G**. (**I**) Mean number of total fibres in TA muscles from 3-month-old control and *Hacd1^−/−^* mice (*n* = 5 for each group). Scale bar, 100 μm. Error bars correspond to standard error of the mean. *P < 0.05, **P < 0.01, ***P < 0.001.

This phenotype was reminiscent of that of *HACD1*-deficient Labrador retrievers (hereafter *HACD1^cnm/cnm^* or CNM dogs) (Tiret et al., 2003; Maurer et al., 2012). As for *Hacd1^−/−^* mice, CNM dogs had reduced weight gain in the first weeks of life compared with controls (Supplementary Figure S2A and B). Postural and locomotor weakness was obvious between 2 and 6 months of age and histological analyses of skeletal muscles from 5-month-old CNM-affected pups revealed that amyotrophy was accompanied by heterogeneity in muscle fibre diameter (Figure 2A and B). Comparative studies between CNM dogs and *Hacd1^−/−^* mice (Figure 2A, B, E, and F) revealed that in both cases, the mean myofibre diameter was reduced by 29.7% and 22.3%, respectively (Figure 2C and G), and the distribution of fibre diameters was shifted towards smaller diameters in comparison with controls (Figure 2D and H); in *Hacd1^−/−^* mice, the number of myofibres per muscle was unchanged (Figure 2I), indicating that fibre hypotrophy *per se* yielded the reduced muscle mass.

These data suggested an early requirement of *HACD1* during muscle development and an important role for muscle fibre growth.

### Hacd1 is required for optimal myoblast fusion

**Figure 3:**
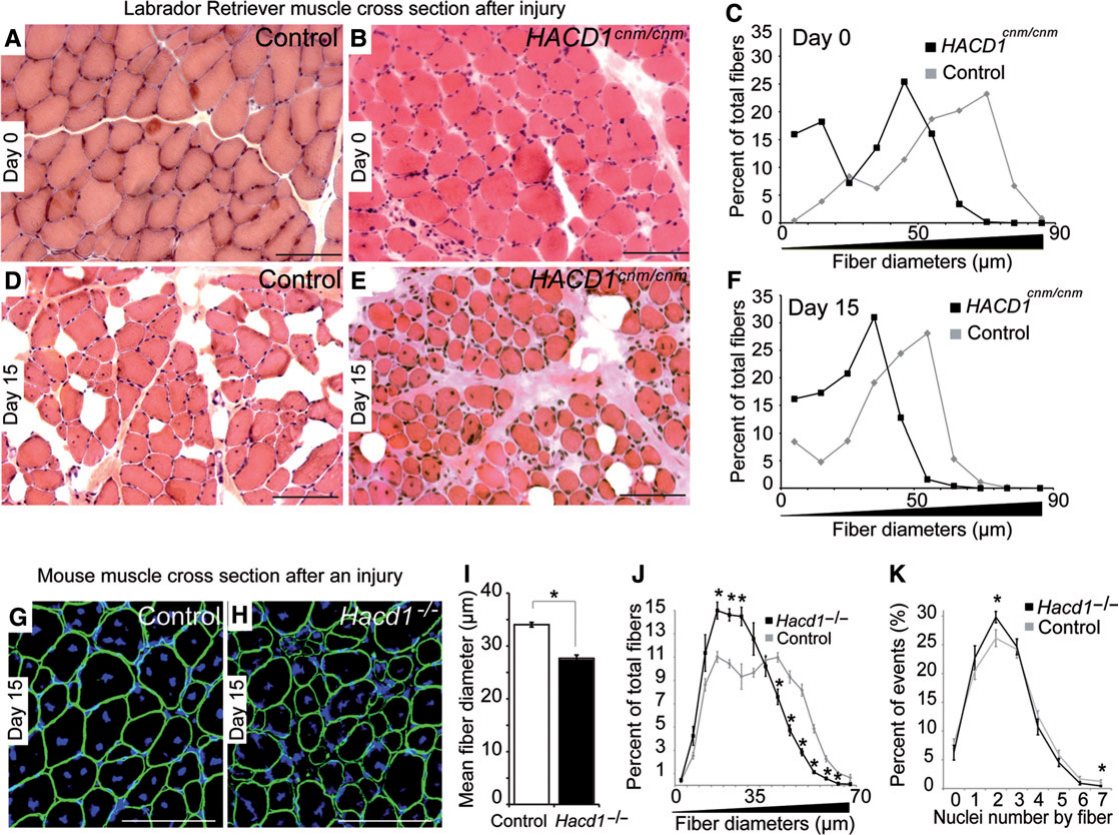
Defective myoblast fusion in HACD1-deficient dogs and mice. H&E-staining of muscle transverse sections before (**A** and **B**) and on Day 15 after notexin injection (**D** and **E**) in control (**A** and **D**) and *HACD1^cnm/cnm^* (**B** and **E**) dogs. Scale bar, 200 μm. (**C** and **F**) Percent distribution by diameter (μm) of myofibres from dog muscles before (**C**) and on Day 15 after notexin injection (**F**) (*n* = 1 for each genotype; ≥730 fibres examined per dog). (**G** and **H**) Immunofluorescence for Dystrophin (green) on TA sections on Day 15 after notexin injection in control (**G**) and *Hacd1^−/−^* (**H**) mice. Nuclei are in blue. Scale bar, 100 μm. (**I**) Mean fibre diameter in TA muscles on Day 15 after notexin injection in control and *Hacd1^−/−^* mice (*n* = 3 and 4, respectively; ≥1780 fibres per mouse). (**J**) Percent distribution by diameter (μm) of myofibres analysed in **I**. (**K**) Nuclear content of fibres (*n* = 3 for each group; ≥600 fibres per mouse). Error bars correspond to standard error of the mean. *P < 0.05, **P < 0.01, ***P < 0.001.

In order to characterize the cause of the reduced muscle mass and further discriminate between muscle atrophy and hypotrophy, we recapitulated myofibre development during muscle regeneration in both dogs and mice. The course of regeneration was similar in controls, CNM dogs and *Hacd1^−/−^* mice. Four to six days after notexin-induced injury, massive muscle necrosis and cellular infiltration were observed (Supplementary Figures S1G and S2C), replaced on Day 15 by regenerating myofibres (Figure 3A, B, D, E, G, H, and Supplementary Figure S2C). However, newly formed myofibres were already significantly hypotrophic and the largest myofibres were almost completely absent in CNM dogs and *Hacd1^−/−^* mice (Figure 3C, F, I, and J), identifying myofibre hypotrophy as a pathogenic mechanism. This reduced diameter of fibres was still present on Day 30 and 90 of regeneration in CNM dogs (Supplementary Figure S2C), indicating that no compensatory growth overcame this developmental defect. It is well-accepted that a deficit in muscle fibre growth observed at this stage of regeneration results from a defect in myoblast fusion (Horsley et al., 2001; Doherty et al., 2005; Georgiadis et al., 2007; Hochreiter-Hufford et al., 2013; Lenhart et al., 2014; Millay et al., 2014). Accordingly, nuclear counts on Day 15 confirmed that myofibres containing two nuclei were over-represented in *Hacd1^−/−^* mice compared with controls, whereas myofibres with 7 nuclei (the highest number counted at this stage) were depleted (Figure 3K).

**Figure 4:**
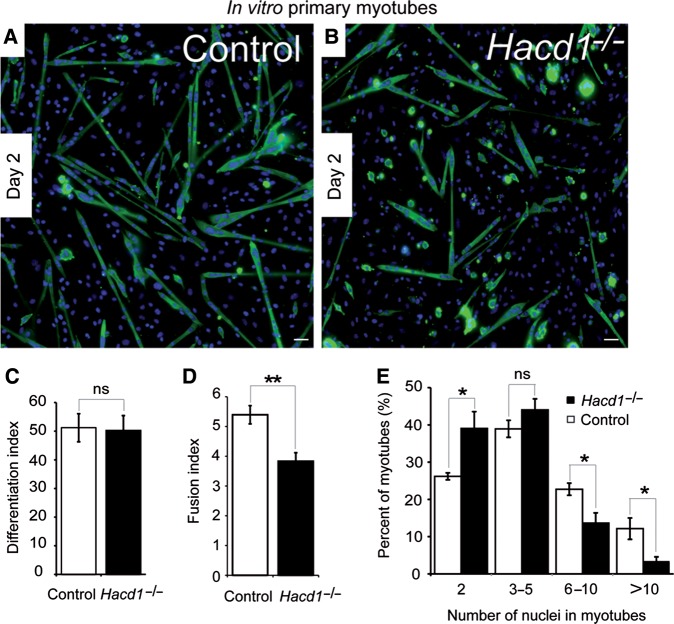
Defective myoblast fusion in normally differentiating HACD1-deficient myoblasts. (**A** and **B**) Immunodetection on muscle primary cell cultures from control or *Hacd1^−/−^* newborns on Day 2 of differentiation of the myosin heavy chains (MHC, green). Nuclei are in blue. Scale bar, 100 μm. (**C**) Unchanged differentiation index in HACD1-deficient myoblasts, i.e. percentage of nuclei contained in MHC-positive cells (*n* = 5 for each newborn group; ≥1000 nuclei per sample). (**D**) Decreased fusion index in HACD1-deficient myoblasts, i.e. average number of nuclei per myotube (*n* = 5 for each newborn group; ≥100 myotubes per sample). (**E**) Percent distribution of myotubes analysed in **D** by their number of nuclei. Error bars correspond to standard error of the mean. *P < 0.05, **P < 0.01, ***P < 0.001.

To further confirm the impairment of myoblast fusion and determine if this defect was combined with a depletion in satellite cells, we first assessed in mice muscles the number of *Pax7*-positive satellite cells from which myoblasts differentiate to form new myofibres. Both immunofluorescence and RT-qPCR experiments indicated a similar expression of *Pax7* in *Hacd1^−/−^* mice compared with controls (Supplementary Figure S3A–D), excluding reduced numbers of satellite cells as causative of hypotrophy. We then used the capacity of primary myoblasts isolated from young pups to differentiate *in vitro* to characterize the contribution of *Hacd1* in fusion. After 2 days of differentiation, cells isolated from control and *Hacd1^−/−^* pups similarly formed myotubes and the number of nuclei within myosin heavy chain-positive cells was similar, indicating normal myoblast commitment into the differentiation process (Figure 4A–C). In contrast, myotubes were globally smaller in cultures from *Hacd1^−/−^* pups and presented a reduced fusion index assessed by the average number of nuclei per myotube (Figure 4A, B, and D). Accordingly, distribution of myotubes with respect to their number of nuclei revealed fewer myotubes with ≥6 nuclei, and greater numbers of small myotubes with only 2 nuclei compared with controls (Figure 4E).

Together, these results demonstrate that both in dogs and mice, *Hacd1* function triggers the optimal fusion of myoblasts during their differentiation, sustaining the normal growth of muscle fibres within muscles.

### The muscle-enriched Hacd1-fl isoform is upregulated during muscle development and regeneration

**Figure 5:**
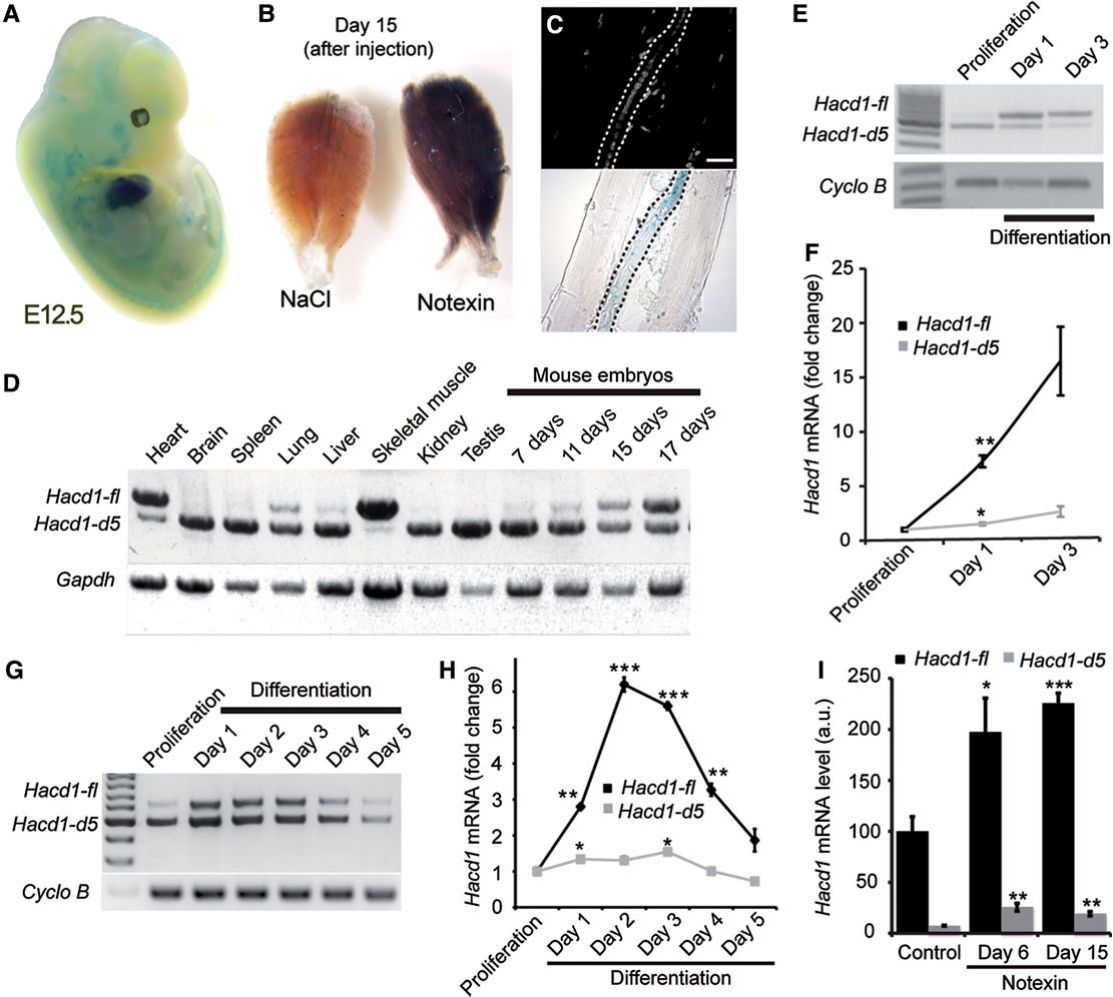
Upregulation of the muscle-specific, *Hacd1-full length* (*Hacd1-fl*) isoform during muscle fibre development. (**A** and **B**) X-Gal staining of a *Hacd1^+/−^* embryo at E12.5 (**A**), TA muscles (**B**) from a *Hacd1^−/−^* mouse on Day 15 after injection of NaCl (left) or notexin (right). (**C**) Dissected fibres from the notexin-injected muscle shown in **B** showing X-Gal staining in a regenerative fibre (centralized nuclei, in blue). Scale bar, 20 μm. (**D**) RT-PCR experiments showing expression of *Hacd1* transcripts in mouse tissues and embryos. (**E**–**H**) Expression of *Hacd1* transcripts during differentiation of primary muscle cells (**E** and **F**) and C2C12 cells (**G** and **H**) revealed by RT-PCR experiments (**E** and **G**) or RT-qPCR experiments (**F** and **H**) showing fold-change from proliferation for each isoform (*n* = 3 for each condition). (**I**) RT-qPCR experiments for *Hacd1-fl* and *Hacd1-d5* isoforms in TA muscles from wild-type mice, either non-injected (*n* = 4) or on Day 6 (*n* = 3) or Day 15 (*n* = 4) after notexin injection. Error bars correspond to standard error of the mean. *P < 0.05, **P < 0.01, ***P < 0.001.

During embryogenesis of *Hacd1^+/−^* mice, strong expression of the *LacZ* reporter gene was observed in heart and skeletal muscle precursors (Figure 5A). *LacZ* expression was also highly induced in adult skeletal muscles during injury-induced regeneration (Figure 5B) and, more precisely, in regenerating myofibres recognizable by the alignment of central nuclei (Figure 5C).

In wild-type dogs, two *HACD1* splice variants are expressed in normal skeletal muscles: the *HACD1-fl* full-length isoform retains the seven exons of the gene whilst the *HACD1-d5* isoform lacking exon 5 encodes a truncated protein (Pelé et al., 2005). RT-PCR conditions designed to amplify both variants revealed a broad expression of the *Hacd1-d5* isoform in mouse tissues (Figure 5D). In contrast, *Hacd1-fl* expression was nearly restricted to the heart and skeletal muscles, and in these tissues it was the predominant, highly-expressed isoform. During mouse embryonic development, a shift in *Hacd1* splicing occurred between Day 7 and 17, with a marked increase in *Hacd1-fl* expression at the expense of *Hacd1-d5* (Figure 5D). This embryonic period spans muscle fibre maturation (Buckingham, 2001). To evaluate whether these two variants would play specific dynamic roles during muscle growth, we investigated by RT-(q)PCR their temporal expression during *in vitro* differentiation of wild-type mouse primary or normal C2C12 myoblasts. We observed that formation of myotubes followed a marked upregulation of *Hacd1-fl* expression whereas *Hacd1-d5* expression was only mildly increased or did not change in both primary myoblasts (Figure 5E and F) and C2C12 (Figure 5G and H). *In vivo*, *Hacd1* was also markedly induced during muscle regeneration, strengthening *Hacd1-fl* predominance over *Hacd1-d5* in muscles (Figure 5I). Expression of *Hacd2* and *Hacd3* did not change during differentiation (Supplementary Figure S3E).

### HACD1-FL is necessary for myoblast fusion

**Figure 6:**
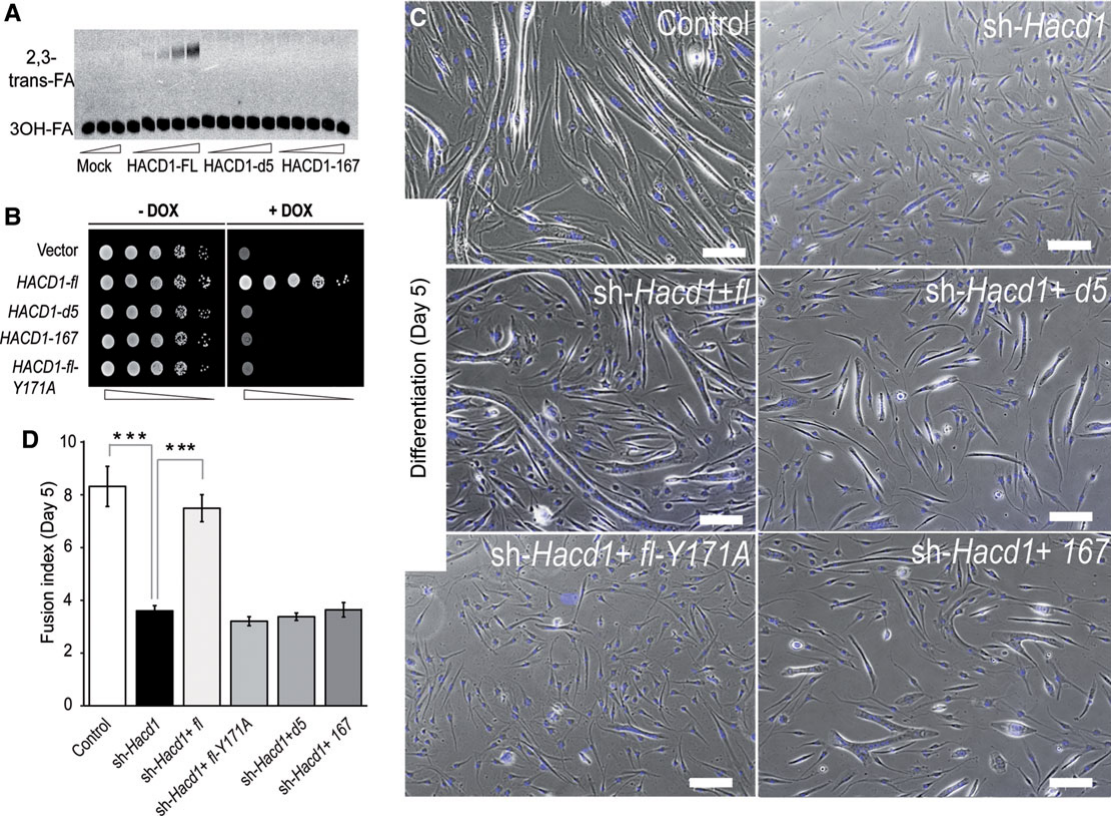
Activity of HACD1 isoforms and role of *HACD1-fl* in myoblast fusion. (**A**) Affinity-purified 3×FLAG-tagged HACD1 isoforms incubated with [^14^C]3-hydroxypalmitoyl-CoA (3-OH FA) and separated by normal-phase thin layer chromatography. (**B**) Shutdown of *PHS1* in a yeast strain carrying *PHS1* under control of a tetracycline (doxycycline; DOX)-dependent promoter leads to a lethal phenotype, rescued only by the active wild-type *HACD1-fl* isoform. Cells were serially diluted at 1:10. (**C**) On Day 5 of differentiation, sh-*Hacd1* cells failed to form elongated myotubes, a phenotype rescued only by re-expression of the wild-type *HACD1-fl* (sh-*Hacd1*+*fl*) isoform. Scale bar, 200 μm. (**D**) Fusion index of sh-*Hacd1* cells and sh-*Hacd1* cells re-expressing HACD1 isoforms (*n* = 3 for each condition).

HACD1-FL protein is a vertebrate ortholog of the yeast enzyme Phs1 and displays HACD activity (Ikeda et al., 2008). In addition, we reported above a broad expression of *Hacd1-d5* in mouse tissues; besides, *HACD1-167*, an aberrant splice variant retaining exons 1, 6, and 7, specifically accumulated in muscles from CNM dogs (Pelé et al., 2005). We thus investigated whether the two HACD1-D5 and HACD1-167 proteins encoded by these isoforms, predicted respectively to exhibit two or three transmembrane domains instead of the six identified in HACD1-FL (Supplementary Figure S4A), shared cellular and molecular features with HACD1-FL. In myogenic cells, all three isoforms correctly localized to the ER. In addition, HACD1-FL, HACD1-D5, HACD1-167, as well as HACD2, HACD3, and HACD4 physically interacted with all other proteins of the elongation complex, with the exception of HACD1-D5 that did not interact with TER (Supplementary Figure S4B–D). *In vitro*, enzymatic assays revealed that HACD1-FL, but neither HACD1-D5 nor HACD1-167, catalysed the dehydration of 3-hydroxypalmitoyl-CoA into 2,3-*trans*-hexadecenoyl-CoA, in a dose-dependent manner (Figure 6A and Supplementary Figure S4E). Accordingly, *in vivo* expression of HACD1-FL, but not HACD1-D5, HACD1-167, or HACD1-FL-Y171A in which the Y171 essential residue (Kihara et al., 2008) had been mutated, rescued cell growth arrest of *PHS1*-shutdown yeasts (Figure 6B and Supplementary Figure S4F).

To decipher the relative functional importance of HACD1 isoforms during muscle cell differentiation, we stably knocked down *Hacd1* expression in C2C12 cells using a small hairpin interfering RNA targeting the wild-type *Hacd1-fl* and *Hacd1-d5* isoforms (Supplementary Figure S5A). Myotube formation was severely impaired in *Hacd1-*knocked down (sh*-Hacd1)* cells (Figure 6C), confirming previous data (Lin et al., 2012). Defective fusion events were highlighted by a severe reduction in the number of nuclei per sh*-Hacd1* myotube, compared with controls (Figure 6C and D), although commitment of sh*-Hacd1* cells into the differentiation process was confirmed by increased expression of the *Myogenin* and *Myosin, heavy polypeptide 2, skeletal muscle, adult* (*Myh2*) myogenic markers (Supplementary Figure S5B–D) in line with the phenotype of the *Hacd1^−/−^* myoblasts (Figure 4A and B). This phenotype was thus consistent with our *in vivo* experiments that revealed an impairment in myoblast fusion upon HACD1 deficiency.

To evaluate the function of each isoform, we transduced sh*-Hacd1* cells with retroviral vectors driving the expression of shRNA-insensitive sequences encoding each of them (sh*-Hacd1+isoform* cells) (Supplementary Figure S5E and F). Importantly, rescue of the fusion impairment could be elicited only when the catalytically active HACD1-FL isoform was expressed, demonstrating the essential role of its specific activity in myoblast fusion (Figure 6C and D).

In parallel, *sh-Hacd1* cells exhibited a reduced proliferation rate (data not shown), as previously reported with the same interfering sequence (Lin et al., 2012). However, in our hand, this phenotype was neither rescued by HACD1-FL nor by HACD1-D5 and hence likely resulted from an off-target effect of the RNA interference. The fact that HACD1-FL could rescue the fusion defect without rescuing the proliferation defect further indicates that the proliferation rate did not interfere with the fusion capacity of sh-*Hacd1* cells.

### HACD1-FL regulates lipid balance and membrane fluidity

To identify the putative HACD-dependent changes in lipid content during myoblast differentiation, we compared phospholipid fatty acid contents in proliferative C2C12 myoblasts, when the *Hacd1-fl* to *Hacd1-d5* ratio was the lowest, with those on Day 3 of differentiation, corresponding to 1 day after the peak of *Hacd1-fl* expression (Figure 5G, H and Supplementary Figure S3E).

**Figure 7:**
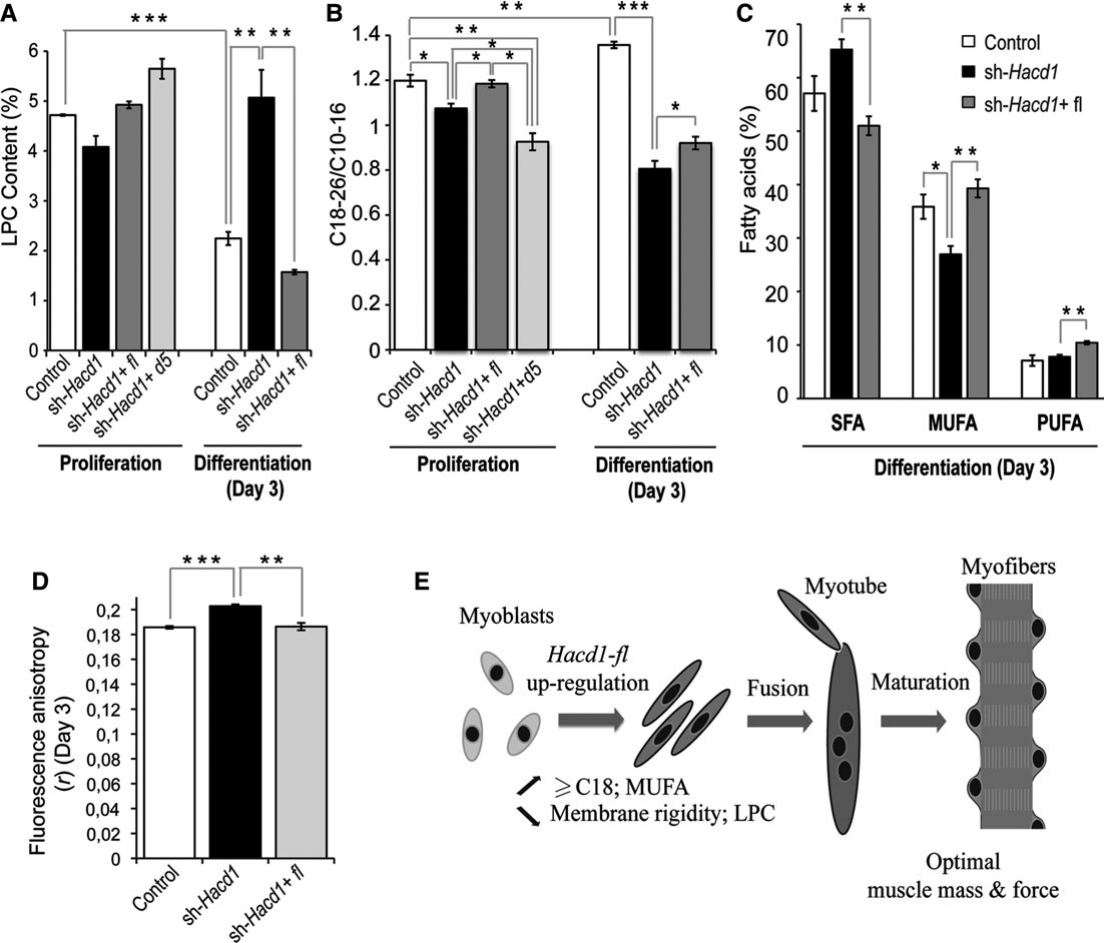
HACD1 regulates lipid balance and membrane fluidity in myoblasts. (**A**) Lysophosphatidylcholine (LPC) content, expressed as the percentage of total phospholipids, in proliferation and on Day 3 of differentiation. (**B**) Ratio of C18-26 to C10-16 phospholipid fatty acids in proliferation and on Day 3 of differentiation. (**C**) Proportions of saturated (SFA), monounsaturated (MUFA), or polyunsaturated (PUFA) fatty acids on Day 3 of differentiation. (**D**) Fluorescence anisotropy (*r*, inverse of fluidity) measured on Day 3 of differentiation. (**E**) Proposed model for the role of *Hacd1-fl* during muscle fibre development. Error bars correspond to standard error of the mean. *P < 0.05, **P < 0.01, ***P < 0.001.

As lysophosphatidylcholine (LPC) acts as a potent inhibitor of myoblast fusion when added to the differentiation medium (Leikina et al., 2013), we first evaluated whether the relative content of phospholipid species would be modified upon *Hacd1* modulation (Supplementary Table S2). In control myoblasts, differentiation was accompanied by a nearly two-fold drop in LPC that was abolished in sh*-Hacd1* myoblasts and fully restored in sh*-Hacd1+*FL cells (Figure 7A and Supplementary Table S2A). This global dynamic profile was underlain by the individual profile of most LPCs (Supplementary Table S2B). This result demonstrated that HACD1-FL activity is responsible for the drop in LPC content preceding fusion. Myoblast differentiation was also accompanied by an increase in the level of phosphatidylinositols and a decrease in lysophosphatidylethanolamines that were independent of *Hacd1* expression (Supplementary Table S2A).

In parallel, we evaluated fatty acid content in the whole phospholipid pool (Supplementary Table S3). As C18 species are the smallest fatty acids produced by the VLCFA elongation complex (Ohno et al., 2010), the ratio of C18-C26/C10-C16 fatty acids reflected the elongation efficiency. Compared with controls, the ratio was decreased in sh*-Hacd1* cells both in proliferation and in differentiation, and this elongation defect was alleviated by HACD1-FL re-expression whereas it was amplified following HACD1-D5 expression (Figure 7B and Supplementary Table S3A and B). These results confirmed a positive role of HACD1-FL in the elongation of ≥C18 fatty acids and revealed an antagonistic role for HACD1-D5. Accordingly, the increase in the *Hacd1-fl* to *Hacd1-d5* ratio reported above in differentiating control myoblasts was accompanied by an increase in the C18-C26/C10-C16 ratio (Figure 7B and Supplementary Table S3B).

Interactions between the yeast desaturase Ole1 and the VLCFA elongation complex have been described (Miller et al., 2005). To evaluate whether such an interaction could be conserved and functional in mammals, we investigated the saturation status of phospholipid fatty acids in myoblasts. Indeed differentiating sh*-Hacd1* myoblasts had an increased saturated fatty acid (SFA) content, at the expense of monounsaturated fatty acids (MUFA) (Figure 7C and Supplementary Table S3C). Re-expression of HACD1-FL fully restored normal SFA and MUFA contents and slightly increased the polyunsaturated fatty acid (PUFA) content. During proliferation too, HACD1-FL promoted MUFA accumulation at the expense of SFA and HACD1-D5 isoform exhibited an antagonist role (Supplementary Figure S6A and Table S3C).

Unsaturation of fatty acids contained in phospholipids is known to increase fluidity of lipid bilayers (Maxfield and Tabas, 2005). To check whether the altered contents of SFA, MUFA, and PUFA could have a functional impact on myoblasts, we analysed their plasma membrane fluidity on Day 3 of differentiation and found that it was reduced by 9% in sh*-Hacd1* cells at 37°C (*P* = 5.10^−5^). Fluidity was fully restored by HACD1-FL re-expression (Figure 7D and Supplementary Figure S6B).

## Discussion

We showed that HACD1-deficient mice and dogs exhibit an early-onset hypotrophy of myofibres that could be recapitulated during muscle regeneration after injury, two characteristics of myoblast fusion impairment (Horsley et al., 2001; Doherty et al., 2005; Georgiadis et al., 2007; Laurin et al., 2008; Hochreiter-Hufford et al., 2013; Millay et al., 2013, 2014; Lenhart et al., 2014). Defective myoblast fusion upon HACD1 deficiency was confirmed both *in vivo* and *in vitro*, as indicated by smaller myofibres containing fewer nuclei. Such an early phenotype could be the result of a defect either in myoblast differentiation or in myoblast fusion *per se*. On the contrary to previously published data (Lin et al., 2012), we observed a normal commitment of both primary myoblasts and C2C12 cells into the differentiation upon *Hacd1* deficiency; in both cases, we reproducibly showed at the RNA and protein levels that *Hacd1* deficiency neither impaired *Myogenin* and *Myh2* expression nor reduced the proportion of cells positive for MHC, a marker of terminal differentiation. This demonstrates that myoblast fusion step was more specifically affected by *Hacd1* deficiency.

On the molecular level, we showed that during myoblast differentiation, HACD1-FL activity prompts a drop in the content of LPC, an inhibitor of myoblast fusion (Leikina et al., 2013). In parallel, it promoted a slight increase in ≥C18 fatty acids, a rise in MUFA content and a drop in plasma membrane rigidity. We propose a model in which HACD1-FL upregulation during myoblast differentiation modifies membrane lipid balance and its physical properties to promote efficient fusion, eventually leading to optimal muscle mass and force (Figure 7E).

In CNM dogs (Pelé et al., 2005), exchange of wild-type *HACD1* transcripts by those of the *HACD1-167* isoform (which encodes an inactive protein), mimics the human condition in which a non-sense mutation abrogates the HACD activity of the encoded protein (Muhammad et al., 2013). As the presence of small myofibres is also observed in HACD1-deficient humans, we suggest that defective myoblast fusion constitutes a novel, non-exclusive pathological mechanism that participates to the reduction in muscle mass and strength observed upon HACD1 deficiency. More generally, myofibre heterogeneity with hypotrophic fibres is an early feature of other myopathies with congenital fibre size disproportion and CNM/MTM myopathies (Jungbluth et al., 2008; North, 2008; Nance et al., 2012). Importantly, small fibre diameter, but not the proportion of central nuclei, is associated with the most severe *MTM1* mutations and constitutes the worst outcome in myotubular patients (Pierson et al., 2007). Hence we propose that defective myoblast fusion might constitute a synergistic pathogenic mechanism operating in these congenital myopathies (Ravenscroft et al., 2015). If confirmed, a fusion defect would have direct medical consequences, as it will be mandatory to envisage its early correction within the very first period of postnatal life. Additional mechanisms underlying the accompanying concomitant development of hypertrophic fibres in some cases of myopathies with congenital fibre size disproportion (Jungbluth et al., 2008; Muhammad et al., 2013), would also need to be assessed. Importantly, no major cardiovascular phenotype could be observed in HACD1-deficient young adult dogs and mice, which heart and cardiovascular parameters were thoroughly evaluated using electrocardiography, arterial blood pressure measurement, echocardiography, and tissue-Doppler imaging (data not shown). Of note, in contrast to skeletal muscles, heart development does not rely on cell fusion events. This developmental difference may contribute to protecting the heart from morbid consequences of HACD1 deficiency; it also highlights the pathogenic role of fusion impairment in skeletal muscles.

Changes in membrane fluidity and the degree of saturation of membrane fatty acids each influence myoblast fusion (Prives and Shinitzky, 1977; Nakanishi et al., 2001; Briolay et al., 2013). Our results emphasize that *Hacd1* may play a pivotal role in the integrated genetic control of these previously reported changes, as well as in the newly identified drop in LPC level. During myofiber development, *Hacd1-fl* is upregulated and counteracts the *Hacd1-d5* ubiquitous isoform that exerts an antagonistic role through a still unknown mechanism. As a direct consequence of its 3-hydroxyacyl-CoA dehydratase activity in the VLCFA elongation complex (Ikeda et al., 2008), HACD1-FL promoted an increase in the C18-C26 to C10-C16 ratio during myoblast differentiation. Our data also revealed indirect key functions of *Hacd1* that were not predicted by its HACD activity, which consist in an increased MUFA content at the expense of SFA content and in a drop in both membrane rigidity and LPC content. While there are growing evidences of structural and functional interactions between enzymes of different lipid pathways that we did not investigate in this study, our results suggest a significant interplay of these pathways during myoblast fusion and indicate that *Hacd1* plays a key role in their integrated regulation. These results also open novel medical avenues, as manipulation of the complex *Hacd1*-dependent lipid balance may represent an alternative, non-invasive therapeutic strategy in *HACD1*-related or other congenital myopathies.

Although these changes might influence different steps of myoblast fusion such as myoblast migration or membrane merging, they are all expected to favour membrane fusion *per se*. First, LPC has been reported to inhibit several events of membrane fusion (Reporter and Raveed, 1973; Chernomordik et al., 1993; Yeagle et al., 1994; Ciechonska and Duncan, 2014), presumably because its inverted cone-shape interferes with the negative membrane curvature required for the hemifusion step (Chernomordik and Kozlov, 2005). Accordingly, LPC can reversibly inhibit myoblast fusion, although its effect may depend upon its concentration and the stage of fusion (Leikina et al., 2013; Teng et al., 2015). Our data showed that *Hacd1* regulates the LPC drop normally observed during myoblast differentiation. We suggest that this drop in total membranes increases myoblast permissiveness to fusion and that the failure of this drop observed in *Hacd1*-deficient cells constitutes a major determinant of myoblast fusion impairment in HACD1-deficient conditions. Whether this LPC drop is of significant importance for specific membrane compartments, including the plasma membrane, will require further analyses. In parallel, longer acyl chains of VLCFAs are proposed to fill the voids created during membrane bending and to stabilize strong membrane curvatures (Schneiter et al., 2004; Chernomordik and Kozlov, 2005). Like other fusion processes, myoblast fusion involves a hemifusion stage for plasma membranes, during which lipids from the two merging outer layers are mixed (Chernomordik and Kozlov, 2005; Leikina et al., 2013), followed by the formation of a fusion pore inducing cell content mixing. These two sequential steps necessitate strong membrane curvature that would be favoured by VLCFAs, in accordance with the defective vesicle fusion observed during cytokinesis in plants lacking VLCFA-containing sphingolipids (Molino et al., 2014). Finally, membrane fluidity is a well-known determinant of successful membrane fusion (Maxfield and Tabas, 2005) and in myoblasts, it could also promote fusion by concentrating fusion-associated factors or lipid rafts at sites of contact between activated myoblasts (Abmayr and Pavlath, 2012). During myoblast differentiation, we showed that *Hacd1* activation yields increased levels of MUFAs at the expense of SFAs, a mechanism that likely participates to the increased membrane fluidity and would explain the enhanced myoblast fusion reported following addition of unsaturated fatty acids, whereas saturated fatty acids or cholesterol, a membrane rigidifier, tend to inhibit fusion (Prives and Shinitzky, 1977; Nakanishi et al., 2001; Lee et al., 2009; Briolay et al., 2013).

In conclusion, the results presented here provide a unifying genetic control accounting for several changes in lipid balance that likely confer permissiveness for myoblast fusion in an additive manner. This promoting function would result from the increase in *Hacd1-fl* expression following a muscle-enriched splicing mechanism. In addition to increasing the VLCFA content through its HACD enzymatic activity, HACD1-FL would modify MUFA and LPC levels indirectly, by regulating their respective biosynthetic pathways. Desaturation of SFAs into MUFAs mostly relies on the desaturase activity of ER-resident stearoyl-CoA desaturase proteins (Guillou et al., 2010), and in yeast, physical interactions described between the desaturase Ole1 and proteins of the VLCFA elongation complex (Miller et al., 2005) suggest functional cross-talk between the two enzymatic pathways. The LPC level results from a more complex mechanism since it can be generated either by the cleavage of phosphatidylcholine by phospholipase A2 or by the lecithin-cholesterol acyl-transferase activity. Interestingly, it has been shown that increased LPC synthesis constitutes an important step leading to atherosclerosis (Schmitz and Ruebsaamen, 2010). LPC is also released by several tumour cell lines and because of autotaxin activity, generates the potent cell motility activator lysophosphatidic acid (Gotoh et al., 2012). Thus, identification of splicing factors that upregulate *Hacd1-fl* and reduce LPC expression, which is an issue in developmental myology, might be of wider medical interest.

Further studies will be required to precisely assess the role of *Hacd1* on the metabolism, subcellular localization and function of these candidate lipids, as well as on the indirect modulation of pathways regulating LPC and unsaturated fatty acids contents.

## Materials and methods

### Mice and myoblasts

The *Hacd1* knockout recombination vector (PRPGS00067_A_B09) was obtained from the Knockout Mouse Project Repository (KOMP; https://www.komp.org) and was electroporated into C57BL/6N ES cells. All experiments were performed on mice generated from two independent ES clones. Matings between *Hacd1^+/−^* mice generated control (wild-type and *Hacd1^+/−^*) and *Hacd1* knockout (*Hacd1^−/−^*) mice for all the described experiments.

The ANSES/EnvA/Upec Ethics Committee (C2EA – 16; www.enseignementsup-recherche.gouv.fr) approved the experiments performed on mice (approval numbers 11/11/15-2 and 20/12/12-16).

sh*-Hacd1* and control myoblasts were obtained by selecting puromycin-resistant clones of C2C12 myoblasts, respectively transfected with two different shRNA pGIPZ lentiviral vectors against *Hacd1* exon 4 or a control vector (OpenBiosystems).

### Statistical analysis

A *t*-test-ANOVA with repeated measure factor was applied to paired data, i.e. in the case of measurement of fibre number, size, nuclear content, or PAX7-positive cell content on muscle sections, as well as mass measurements of paired muscles in mice. Student's *t*-test was used for all other analyses and Welch modification was applied when sample number was low (*n* < 4). Data are expressed as mean ± standard error of the mean and differences were considered significative when *P* < 0.05.

### Experimental details

Experimental details of regeneration experiments in dogs and mice; immunological and histological staining; muscle section analyses; tetanic force measurement; PCR, RT-PCR, and RT-qPCR experiments; fluidity; HACD activity measurements; phospholipid analyses; co-affinity purification experiments; additional ethics statement are available in the Supplementary material.

## Supplementary material

Supplementary material is available at *Journal of Molecular Cell Biology* online.

## Funding

This work was supported by the Agence Nationale de la Recherche (ANR-12-JSV1-0005), the Association Française contre les Myopathies (14577, 15882, and 16143), the CNM Project (www.labradorcnm.com), the Alliance program (22866ZM), the Myotubular Trust and Grants-in-Aid for Scientific Research (B) to A.K. from Japan Society for the Promotion of Science (23370057). J.B. was supported by the French Ministry of Research and Technologies and the Université Paris 6 (Paris), V.G., A.P., and A.R. were supported by the ANR, N.B-G. and I.B. were supported by the AFM, and G.W. was supported by the BBSRC CASE and the Myotubular Trust.

**Conflicts of interest:** none declared.

## Supplementary Material

Supplementary Data
