# Supplementary material for: HACD1, a regulator of membrane composition and fluidity, promotes myoblast fusion and skeletal muscle growth
Source: J Mol Cell Biol. 2015 Jul 9;7(5):429–40. doi: 10.1093/jmcb/mjv049 (PMC4589950; doi:10.1093/jmcb/mjv049)
Supplement: Supplementary Data [file supp_7_5_429__index.html]

HACD1, a regulator of membrane composition and fluidity, promotes myoblast fusion and skeletal muscle growth — HACD1, a regulator of membrane composition and fluidity, promotes myoblast fusion and skeletal muscle growth — Supplementary Data 

# *HACD1*, a regulator of membrane composition and fluidity, promotes myoblast fusion and skeletal muscle growth

## Supplementary Data

Supplementary Data

- Supplementary Data - Pdf file
