# Supplementary material for: HACD1, a regulator of membrane composition and fluidity, promotes myoblast fusion and skeletal muscle growth
Source: J Mol Cell Biol. 2015 Jul 9;7(5):429–40. doi: 10.1093/jmcb/mjv049 (PMC4589950; doi:10.1093/jmcb/mjv049)

# Supplementary material

## Animals, Materials and Methods

### Generation of *Hacd1* knockout mice

The *Hacd1* knockout recombination vector (PRPGS00067_A_B09) was obtained from the Knockout Mouse Project Repository (KOMP) (https://www.komp.org/). The targeting vector carried *loxP* sites upstream of exon 2 and downstream of exon 4 of *Hacd1* gene and a *NeoR* cassette flanked by two *loxP* sites upstream of exon 2. In order to generate ES cells heterozygous for the targeting vector, the construct was linearized using AsiSI (New England BioLabs), then purified. The vector was electroporated into C57BL/6N ES cells and G418-resistant clones were screened for homologous recombination by PCR (Long Range PCR, dNTPack (Roche)) using the following primers (Forward primer: 5'ATG GGT GCC TAT TTT CAG TCA GTC A-3'; Reverse primer: 5'AAC TGG TTC CTT CAC GAC ATT CAA C-3'). A second PCR was performed to control the presence of *loxP* site upstream of exon 5 (Forward primer: 5'-TCT TAG GAA GGA GAT GGC GCA-3'; Reverse primer: 5'-AGC CAG CAG GGC TAT AAA CTG AGA C-3'). Chromosomal integrity of the selected clones was checked by karyotyping and Southern blotting was performed to confirm the presence of a single insertion.

Positive clones were then injected into BALB/c blastocysts and chimeric males were mated with C57BL/6N females. Presence of the *Hacd1* targeting allele in individuals from the progeny was assessed by PCR (Forward primer: 5'-TTC ATG TGAA CAC ATT TCT ATT C-3'; Reverse primer: 5'-TGT CTT TTT CTC TAA GCT CCT C-3') (PCR was performed as explained below for RT-PCR). Mice carrying the *Hacd1* targeting allele were bred with *PGK-Cre* mice to generate *Cre*-positive mice carrying a recombinant, knockout *Hacd1* allele (*Hacd1*$^{+/-}$ mice). *PGK-Cre* transgene was eliminated at the following generation and crosses between *Hacd1*$^{+/-}$ mice generated control (wild type and *Hacd1*$^{+/-}$) and *Hacd1* knockout (*Hacd1*$^{-/-}$) mice for the described experiments. All experiments were performed on mice generated from two independent ES clones.

### Muscle dissection and regeneration experiment in mice

Male mice were euthanized by cervical dislocation and the *tibialis cranialis* (TA) and *gastrocnemius* muscles were dissected and frozen as described below. For regeneration experiments, 15 µl of Notexin (Latoxan) ($10^{-5}$M diluted in 0.9% NaCl) were injected into the left TA, and 15 µl of 0.9% NaCl into the right TA (Day 0) of 10 month-old males. Mice were euthanized on Day+6 or Day+15 following notexin injection and TA muscles were dissected and

either frozen for RT-qPCR or immunofluorescence experiments, or stained in X-Gal solution (see below).

## X-Gal staining of mouse embryos and muscles

A C57BL6/N mouse female crossed with a *Hacd1*$^{+/-}$ male was euthanized by cervical dislocation at stage E12.5 of gestation. The uterus was dissected in PBS and embryos were extracted and fixed for 1 h in 4% formaldehyde in PBS at 4 °C, then rinsed and stained overnight at 33 °C in a solution of PBS containing 1% Tween 20, 4 mM potassium ferrocyanide, 4 mM potassium ferricyanide, 2 mM MgCl2, 0.4 mg/ml X-Gal dissolved in DMSO (all reagents were from Sigma). Embryos were then rinsed in PBS. No staining was observed in *Hacd1*$^{+/+}$ embryos, other than that typically-occurring very lightly in the intestinal tract. The same protocol was applied to TA muscles at Day+6 following notexin injection.

## Maximal muscle force measurement in mice

Tibialis anterior muscles of 6-mo-old males were evaluated by the measurement of in situ isometric muscle contraction in response to nerve stimulation as previously described (Schirwis *et al.*, 2013). Mice were anaesthetised using a pentobarbital solution (ip, 60 mg/kg). Feet were fixed with clamps to a platform and knees were immobilized using stainless steel pins. The distal tendons of muscles were attached to an isometric transducer (Harvard Bioscience) using a silk ligature. The sciatic nerves were proximally crushed and distally stimulated by bipolar silver electrode using supramaximal square wave pulses of 0.1 ms duration. All data provided by the isometric transducer were recorded and analyzed on a microcomputer, using PowerLab system (4SP, AD Instruments). All isometric measurements were made at an initial length L0 (length at which maximal tension was obtained during the tetanus). Responses to tetanic stimulation (pulse frequency from 6.25, 12.5, 25, 50, 100 and 143 Hz) were successively recorded. Absolute maximal tetanic force was determined. Muscle mass (m) was measured to calculate specific maximal force (= absolute maximal force/m). All procedures were performed in accordance with national and European legislations and all experimental protocols have been approved by the French Departmental Direction of Animal Protection (agreement 75-1102).

## Muscle biopsy samples and regeneration experiment in dogs

*Biceps femoris* biopsy samples were collected through an open surgical procedure either once in 5-mo-old or 3- to 5-y-old healthy or *HACD1*$^{cnm/cnm}$ Labrador Retrievers or sequentially before (T0) and 4, 15, 30 and 90 days after the notexin injection in 36- to 48-month-old healthy or CNM

Labrador Retrievers. The venom injection was performed as previously described (Sharp *et al.*, 1993; Wilson *et al.*, 1994).

## Ethics statement

The ANSES/EnvA/Upec Ethics Committee (C2EA – 16; www.enseignementsup-recherche.gouv.fr) approved the experiments performed on mice (approval numbers 11/11/15-2 and 20/12/12-16). Labrador Retrievers were dogs maintained in our research colony, and samples used in this study were frozen samples obtained in the 1990s by one of the co-authors (SB). At the time they were sampled, there was no animal welfare committee at the Ecole nationale vétérinaire d'Alfort; however, SB is a certified veterinarian and was accredited by the Veterinary Division of the French Ministry of Agriculture to perform research on animals.

## Cell culture

HEK 293T cells were grown in Dulbecco's modified Eagle's medium (DMEM; Sigma, St. Louis, MO), supplemented with 10% fetal bovine serum (FBS) and penicillin/streptomycin, seeded in dishes coated with 0.3% collagen respectively. C2C12 myoblasts were grown in DMEM (PAA) containing 4.5 g/l glucose, supplemented with 15% FBS (PAA) and penicillin/streptomycin. Cells were maintained at 37 °C in a saturated humidity atmosphere containing 5% $CO_2$. For the differentiation of C2C12 myoblasts, fetal bovine serum was replaced by 2% horse serum (Biowest) when myoblasts reached 70-80% confluence. For fatty acid supplementation, 10 mM fatty acids (Sigma) stock solution maintained at -20 °C in ethanol in glass tubes, were dissolved in DMEM supplemented with 0.25 mM fatty acid-free BSA (Sigma, A6003) at 37 °C for 5 min under strong agitation. Fatty acid solutions were filtrated through 0.2 µm filters (Dutscher, 146560) and diluted in differentiation medium (DMEM + 2% of horse serum) at a final concentration of 5 µM from the first day of differentiation (D0). The fusion index was calculated as the mean number of nuclei per myotube at day 5 of differentiation. Myotubes were defined as myosin heavy chain-positive cells containing at least 2 nuclei.

Primary myoblasts were obtained by dissection of hindlimb muscles from 5-day-old wild type, *Hacd1*$^{+/-}$ and *Hacd1*$^{-/-}$ pups. Muscles were digested in PBS containing 0.5 mg/ml collagenase (GIBCO 17101) and 3.5 mg/ml dispase (GIBCO 17105) for 2 h at 37 °C. Cell suspension was filtered through a 40 µm cell strainer and pre-plated in DMEM + 15% FBS for 4 h at 37 °C, 5% $CO_2$. Non-adherent myogenic cells were collected and plated in IMDM (GIBCO 31980) + 20% FBS onto Ibidi dishes (Biovalley 80426) coated with collagen (Sigma C7661). Differentiation was triggered by changing the culture media to IMDM + 2% of horse serum for 2 days.

## Generation of sh-*Hacd1* cells

Two shRNA pGIPZ lentiviral vectors designed to target *Hacd1* exon 4 (V2LHS_5923 (GCTCATTACTCACAGTATA) and V2LHS_252516 (CTCATTACTCACAGTATAA)) and a control vector (RHS4349; OpenBiosystems) were transfected into C2C12 cells using Arrest-in (OpenBiosystems). After 48h, transfected cells were selected with puromycin (2 mg/ml; Invitrogen). Individual clones were picked after 10 days of selection and shRNA efficiency was checked by RT-qPCR. Initial experiments conducted in parallel in one V2LHS_5923 and one V2LHS_252516 clone proved phenotypic similarity. Clone V2LHS_252516 clone (named sh-*Hacd1* cells) was then selected for the series of functional experiments. Control cells correspond to cells transduced with the control vector.

## Transduction of *HACD1* isoforms

For expression of shRNA-resistant isoforms in control and sh-*Hacd1* cells, cDNAs from the three canine *HACD1* isoforms were isolated by RT-PCR on mRNA extracted from muscles of wild type (*HACD1-fl* and *–d5* isoforms) and CNM-affected (*HACD1-167* isoform) Labrador Retrievers. Three silent mutations (GCTCATCACACATAGTATA) were introduced into exon 4 of *HACD1-fl* and *HACD1-d5* cDNAs by PCR and a MYC tag was introduced at the 5' end of the 3 isoforms using the following primers. Forward primer for all isoforms: GATCCTCGAGACCATGGCATCAATGCAGAAGCTGATCTCAGAGGAGGACCTGGCTGCACTTATGGCGTCCAGCGAGGAG; Reverse primer for *HACD1-fl* and *HACD1-167*: AGATGCGGCCGCTTAATCATCCTTTTCTACAATCACTTCTC; Reverse primer for *HACD1-d5*: AGATGCGGCCGCTCACCAACAACCCCGACAGGA. PCR products were cloned into the pCR4-TOPO vector (Life Technologies). After sequencing, cDNAs were cloned in the MSCV-IRES-BlasticidinR (MSCV-IRES-bsr) retroviral vector using EcoRI restriction. The *HACD1-fl-Y171A* mutation (ATCATCTTAGCTCCTGTCGGGGT) was obtained by PCR from the *HACD1-fl* shRNA-resistant construct and cloned as described.

Retroviral particles were produced by transient triple transfection of HEK293T cells with XtremeGene 9 reagent from Roche, according to manufacturer's instructions, using a 2:1:1 ratio of MSCV-ires-bsr, LTR-env and CMV-gag-pol (a kind gift of Olivier Albagli). Retroviral supernatants were collected 48 h after transfection and concentrated on Amicon Ultra-15 centrifugal

filter units (100 kDa cut-off, Millipore). C2C12 cells were spinfected by a 1 h centrifugation (300 g) in the presence of retroviral supernatant and 8 μg/ml polybrene (Sigma).

Tranduced cells were selected with blasticidin (5 μg/ml; Invitrogen). Plasmid expression was checked by RT-qPCR and immunostaining.

## Extraction of total RNA, RT-PCR and RT-qPCR analysis

Mouse muscle samples were snap frozen in liquid nitrogen and stored at -80 °C. Total RNAs were isolated from C2C12 myoblasts and mouse samples with RNA Nucleospin Kit (Macherey-Nagel) according to the manufacturer's protocol. Purity of RNAs was assessed by a ratio of absorbance at 260 nm and 230 nm > 1.7. RNA quality was checked on agarose gel. One microgram of RNA was used for reverse transcription with the Maxima First Strand cDNA Synthesis Kit for RT-qPCR (Fermentas). cDNA were amplified using the Maxima SYBR Green qPCR Master Mix (2X) (Fermentas). PCR reactions were performed with Taq DNA Polymerase from MP Biomedicals on a Mastercycle Eppendorf thermocycler. qPCR reactions were performed on a Roche Light Cycler Carousel-based system 2.0 (Roche).

Expression of mouse *Hacd1* and *glyceraldehyde 3-phosphate dehydrogenase* (*Gapdh*) mRNAs were examined by PCR using first-strand cDNAs from various mouse tissues and embryonic stages (Mouse MTC panel I; Clontech, TAKARA Bio, Palo Alto, CA, USA) with primer pairs (for *Hacd1*, Hacd1-For-2/Hacd1-Rev-2; and *Gapdh*, Gapdh-For/Gapdh-Rev). These and the other primers used for quantification of gene expression are listed in Table S1. All PCR and qPCR products were examined qualitatively on agarose gels. All presented RT-qPCR results were normalized to *Tbp1* (*Hacd1* expression during muscle regeneration), *Hprt1* (*Pax7* expression) or *CycloB* (all other cases) gene expression.

## Immunofluorescence and histological staining

Control, sh-*Hacd1* or sh-*Hacd1* cells expressing shRNA-insensitive isoforms were grown for 48 h on a glass coverslip (Menzel-Glaser) and then briefly rinsed three times with PBS (PAA), fixed for 15 min with 4% formaldehyde and rinsed with PBS. Cells were permeabilized for 10 min with PBS-0.1% Triton (Sigma), rinsed 3 times with PBS, and blocked with PBS-1% BSA (Sigma) for 1 h before incubation with antibodies. Primary anti-Calnexin (rabbit polyclonal, Sigma, 1:200), anti-Myc (mouse 4A6, Millipore, 1:200), and anti-myosin heavy chain (mouse MF20, DSHB, 1:500) antibodies were incubated overnight at 4 °C and revealed using secondary Alexa 488 goat anti-rabbit (1:500) and Alexa 555 goat anti-mouse (1:500) (Invitrogen). Nuclei were stained with DAPI (4',6' diamidino-2-phenylindol ) 1:4000 for 10 min at RT and slides were mounted using

Fluorescent mounting medium (Dako). Alternatively, they were mounted in aqueous mountant containing the chromatin stain DAPI (1.5 μg/ml) (Vectorshield).

Muscle biopsy samples from dogs and muscles from mice were snap-frozen in isopentane cooled in liquid nitrogen and stored at −80 °C. Transverse-sections (10 μm thickness) were stained with hematoxylin-eosin (H&E) or in an ATPase staining solution following a preincubation at pH = 9.4 or immunostained as previously described for cells with anti-Pax7 (mouse monoclonal, DSHB, 1:20), anti-V5 epitope (rabbit polyclonal, Abcam, 1:500, anti-Ryr1 (mouse Abcam 3C, 1:100) primary antibodies. For Dystrophin immunostaining, fixation was performed with acetone/methanol v/v at -20 °C for 15 min and immunostaining was pursued with anti-Dystrophin (mouse monoclonal, Novocastra, 1:20) antibody. For both anti-PAX7 and anti-Dystrophin antibodies, Mouse Ig Blocking Reagent (MOM kit, Vector, MFB-2213) was used following the manufacturer's instructions. Alexa 488 or 594 goat anti-rabbit (1:500) and Alexa 488 or 555 goat anti-mouse (1:500) (Invitrogen) secondary antibodies were then used. Images were captured using an Axio Observer Z1 microscope (Zeiss) and analyzed using Photoshop CS3.

Routine muscle histopathological evaluation was conducted on the basis of the H&E staining. Diameter and distribution of myofiber type were examined on ATPase 9.4 sections. Quantification of fiber number, size or nuclear content was performed on 7 randomly-selected fields (dog samples) or 3 sections separated by at least 30 μm (mouse samples). Morphometric quantification of the minimal Feret's diameter was done using the Visilog software (Noesis) and quantification of fiber number and nuclear content was done using ImageJ (1.47v). In dog and mouse samples, 500 to 1000 fibers and 700 to 1500 fibers, respectively, were analyzed for each section.

## Plasmid electroporation into murine skeletal muscle

V5-tagged murine *Hacd1-fl* was generated by direct TOPO TA cloning of RT-PCR products into pcDNA3.1/V5-His TOPO (Invitrogen) (primer sequences Forward: 5'-GTCACCATGGCGTCCAGTGAGGAG-3' Reverse: 5'-GTGTGTGGGAACCACTAAAT-3'). Expression vector was electroporated into *tibialis cranialis* (TA) muscles of 7-wk-old female C57BL/6 mice (2 mice bilaterally) according to (McMahon *et al.*, 2001). Mice were anaesthetised with fentanyl/fluanisone (Hypnorm) and midazolam (Hynovel) and injected intramuscularly with bovine testes hyaluronidase (25 μl/muscle of 0.4 μg/μl solution in sterile saline) using an insulin syringe and 28 gauge needle. After 2 h, anaesthesia was deepened with isofluorane inhalation, and 25 μl plasmid solution (0.5 μg/μl) was injected into the TA prior to transcutaneous electroporation performed by applying a potential difference of 81V across the muscle in 10 x 20 ms pulses at 1 Hz frequency (ElectroSquare Porator ECM 830, BTX). After 6 days, mice were euthanized by cervical

dislocation and the TA muscles were dissected and treated as described in the previous section.

### Yeast strain and media

*S. cerevisiae* strain TH_3237 (*MAT***a** *his3*D*1 leu2*D*0 met15*D*0 ura3*D*0 URA3::CMV-tTA pPHS1::KanMX4-TetO$_7$-CYC$_{TATA}$*) (Mnaimneh *et al.*, 2004) was obtained from Open Biosystems (Huntsville, AL). Cells were grown in synthetic complete (SC) medium (0.67% yeast nitrogen base, 2% D-glucose, and nutritional supplements) but lacking histidine (SC-His) at 30 ˚C.

### Western blot analysis

sh-*Hacd1* cells expressing shRNA-insensitive isoforms were washed in PBS and put in lysis buffer (50 mM HEPES pH 7.4, 150 mM NaCl, 0.5% Triton X-100, 10% glycerol, 1 mM DTT, Complete protease inhibitor cocktail (Roche), 1 mM EDTA) for 30 min on ice. Lysates were sonicated and spun for 10 min at 10,000 g. After determination of protein contents using the Bradford method, 20 µg of clarified lysates were separated by SDS-PAGE. Transfer was done on a PVDF membrane (Millipore). Membranes were blocked with TBS-5% skimmed milk for 1 h at RT and incubated with anti-MYC antibody (Mouse 9E10, Sigma, 1:2000) or mouse anti-α-Tubulin (mouse DM1A, Sigma, 1:5000) in TBS-0.1% Tween overnight at 4 °C. After washing, anti-mouse-HRP antibody (Amersham) was applied for 1 h at RT. After washing, antibody-bound proteins were visualised using the ECL+ kit (Amersham). Acquisition was done using a Fusion Fx5 (Vilber Lourmat). Quantification was performed using Photoshop CS3.

Western blot analysis for yeast proteins was performed as described previously (Kihara and Igarashi, 2002), using the anti-FLAG antibody M2 (1 mg/ml; Stratagene, Agilent Technologies, La Jolla, CA, USA) and anti-Pgk1 antibodies (0.25 mg/ml; Molecular Probes, Life Technologies, Eugene, OR) as the primary antibodies, and HRP-conjugated anti-mouse IgG F(ab')$_2$ fragments (at 1:7500 dilution; GE Healthcare Life Sciences, Buckinghamshire, UK) as the secondary antibodies. Labeling was detected using Pierce Western Blotting Substrate (Thermo Fisher Scientific, Waltham, MA, USA).

### Co-Affinity Purification (Co-AP) experiments in HEK293T

cDNAs for *cHACD1-fl, -d5* and *-167* isoforms were cloned as baits in the pDEST27-GST Gateway vector (Invitrogen). cDNAs for *cHACD1-fl, -d5* and *-167* isoforms, and human *HACD2*, *HACD3*, *HACD4*, *ELOVL1* to *7*, *KAR* and *TER* genes were cloned as preys in the pCE-puro 3XFlag-1 vector. Human *EIF2B1* cDNA was cloned in the pDEST-Myc Gateway vector (gift from Marc Vidal's lab) as a control prey. For each interaction assay, GST-bait and Flag/Myc-prey expression vectors were co-transfected in HEK293T cells using calcium phosphate. Each Flag/Myc-prey vector was also co-

transfected with GST vector alone. After 24 h, cells were harvested and lysed with lysis buffer (50 mM HEPES pH = 7.4, 150 mM NaCl, 0.5% Triton X-100, 10% glycerol, 1 mM DTT, 1 mM EDTA, Complete protease inhibitor cocktail (Roche)) for 20 min on ice. Cell lysates were sonicated and pre-cleared by centrifugation for 10 min at 15,000 g at 4 °C. Pre-cleared lysates were incubated with Immobilized Glutathione beads (Perbio) for 1 h at 4 °C. Beads were then washed extensively three times with lysis buffer and then eluted with ½ lysis buffer and ½ loading buffer 2X (125 mM Tris pH = 7.4, 4% SDS, 20% glycerol, bromophenol blue) at 37 °C for 5 min. After denaturation, 15 µg of pre-cleared lysates and a constant volume of eluates were separated by SDS-PAGE and transferred to a PVDF membrane (Millipore). FLAG-, MYC- and GST-tagged proteins were detected as described above, using mouse anti-FLAG (M2, 1:2000), mouse anti-MYC (9E10, 1:2000) and rabbit anti-GST (1:2000) antibodies (Sigma), respectively. FLAG-/MYC-tagged proteins were first revealed, membranes were then stripped using Restore Western Blot Stripping Solution (Pierce) and GST-tagged proteins were revealed with ECL+ reagent (Amersham). Acquisition was done using Fusion Fx5 (Vilber Lourmat).

## HACD activity assay

The assay was performed using 3-hydroxy[1-$^{14}$C]palmitoyl-CoA (55 mCi/mmol; American Radiolabeled Chemicals, St. Louis, MO, USA) as described previously (Kihara *et al.*, 2008).

## Lipids analyses

*Cell preparation*

C2C12 cells were rinsed using 0.9% NaCl, trypsinated and resuspended with proliferation medium. Cells were centrifuged at 4,000 g for 10 min and pellets were rinsed twice using 0.9% NaCl. Tubes containing dry pellets were filled with non-reactive argon to prevent aerial oxidation of samples and then immediately frozen at -80 °C.

*Reagents*

LCMSMS quality grade solvents were purchased from Fischer Scientific (Illkirch, France). Heptadecanoïc acid, tricosanoic acid, pentafluorobenzyl bromide, N,N'-diisopropylethylamine, potassium hydroxide and BHT were purchased from Sigma Aldrich (Saint-Quentin Fallavier, France). Di-myristoyl phosphatidylcholine (DMPC), 19:0-lysophosphatidylcholine (19:0-LPC), di-myristoyl phosphatidylethanolamine (DPME), di-myristoyl phosphatidylserine (DMPS), d18:1-17:0 sphingomyelin (17:0-SM) were from Avanti Polar Lipids (Coger, Paris, France).

*Quantitation of total fatty acids by GC-MS*

Tissues (ca. 10 mg) were saponified with 1 ml of ethanolic potassium hydroxide (final concentration 0.6 N) containing heptadecanoic acid (10 µg), tricosanoic acid (250 ng) used as internal standards (IS) and 50 mg/l of BHT. Samples were incubated at 56 °C for 45 min under argon. Saponified fatty acids were extracted with 1 ml of HCl 1.2 M and 2 ml of hexane. After evaporation of the organic upper phase under vacuum fatty acids were derivatized to pentafluorobenzyl esters with 5 µl of pentafluorobenzyl bromide, 5 µl of diisopropylbenzylamine and 100 µl of acetonitrile at room temperature for 30 min. Derivatized fatty acids were further extracted with 1 ml of water and 2 ml of hexane. The organic phase was evaporated under vacuum. Finally fatty acids were dissolved in 100 µl prior to GC-MS quantification.

Fatty acids esters (1 µl) were injected in split mode on a HP7890A Gas Chromatograph equipped with an HP7683 injector and a HP5975C Mass Selective Detector (Agilent Technologies). Chromatography was performed using a HP-5MS fused silica capillary column (30 mm x 0.25 mm inner diameter, 0.25 m film thickness, Agilent Technologies). The GC-MS conditions were as follows: carrier gas, helium at a flow-rate of 1.1 ml/min; injector temperature, 250 °C, split mode; oven temperature 140 °C, increased at 5 °C/min to 300 °C, and held for 10 min. The mass spectrometer was operated under negative chemical ionization mode with methane as reactant gaz. The ion source temperature and the quadrupole temperature were 150 °C and 106 °C, respectively.

Quantitation of fatty acids was performed by calculating their relative response ratios to heptadecanoic acid and tricosanoic acid (IS). For this purpose molecular ion responses obtained in negative SIM mode were used.

*Quantitation of PC, SM, PS, PE, PI by LCMS-MS*

Cells were spicked with DMPC (1.323 nmol), 19:0-LPC (1.380 nmol), DMPE (1.398 nmol), DMPS (1.083 nmol), 17:0-SM (1.194 nmol) used as internal standards and total lipids were further extracted according to the method of Folch *et al.* (Folch *et al.*, 1957).

Analysis of phospholipids was conducted by LCMS-MS in MRM mode as previously described (Vial *et al.*, 2014).

## Membrane fluidity assay

Plasma membrane fluidity was estimated by fluorescence anisotropy (*r*) as described (Bastiaanse *et al.*, 1995; Le Borgne *et al.*, 2012). (*r*) measurements were conducted using 1,6 diphenyl 1,3,5 hexatriene (DPH) as a probe. In polarized excitation light, a high *r* value reflects increased rotational lifetime of DPH that happens when DPH orientation is stabilized in less fluid membranes. Cultured cells were rinsed, harvested and centrifuged at 1,000 g for 5 min at 4 °C. The resulting

pellets were resuspended in Opti-MEM® Reduced Serum Medium (Life Technologies, Saint Aubin, France) at a concentration of one million cells per ml. Two milliliters of cell suspension were placed into a 1 cm path length spectroscopic quartz cuvette (VWR International, Limonest, France). The cells were stirred and maintained in a thermostatically controlled chamber. After 6 min at 37 °C, 2 µl of the fluorescent probe DPH (1 mM in tetrahydrofuran) were added to label plasma membranes. $r$ was measured at 37 °C 10 min after addition of the probe; after this first series of measurement, the temperature of the chamber was gradually reduced and anisotropy was measured at 35, 30, 25, 20, 15, 10 and 5 °C. Data were expressed as mean ± s.e.m. of three to five independent experiments.

## Statistical analysis

ANOVA with repeated measure factor was applied to paired data, i.e. in the case of measurement of fiber number, size, nuclear content or PAX7-positive cell content on muscle sections, as well as mass measurements of paired muscles in mice. Student T test was used for all other analyses and Welch modification was applied when sample number was low ($n < 4$). Data are expressed as mean ± standard error of the mean and differences were considered statistically significant when $P < 0.05$.

## Supplementary material references


Bastiaanse EML, Atsma DE, Vandervalk LJM, Vanderlaarse A. Metabolic Inhibition of Cardiomyocytes Causes an Increase in Sarcolemmal Fluidity Which May Be Due to Loss of Cellular Cholesterol. Arch. Biochem. Biophys. 1995; 319: 350–354.

Le Borgne F, Guyot S, Logerot M, Beney L, Gervais P, Demarquoy J. Exploration of Lipid Metabolism in Relation with Plasma Membrane Properties of Duchenne Muscular Dystrophy Cells: Influence of L-Carnitine [Internet]. PLoS ONE 2012; 7[cited 2014 Aug 2] Available from: http://www.ncbi.nlm.nih.gov/pmc/articles/PMC3507830/

Folch J, Lees M, Sloane Stanley GH. A simple method for the isolation and purification of total lipides from animal tissues. J. Biol. Chem. 1957; 226: 497–509.

Kihara A, Igarashi Y. Identification and characterization of a Saccharomyces cerevisiae gene, RSB1, involved in sphingoid long-chain base release. J. Biol. Chem. 2002; 277: 30048–30054.

Kihara A, Sakuraba H, Ikeda M, Denpoh A, Igarashi Y. Membrane topology and essential amino acid residues of Phs1, a 3-hydroxyacyl-CoA dehydratase involved in very long-chain fatty acid elongation. J. Biol. Chem. 2008; 283: 11199–11209.

McMahon JM, Signori E, Wells KE, Fazio VM, Wells DJ. Optimisation of electrotransfer of plasmid into skeletal muscle by pretreatment with hyaluronidase -- increased expression with reduced muscle damage. Gene Ther. 2001; 8: 1264–1270.

Mnaimneh S, Davierwala AP, Haynes J, Moffat J, Peng W-T, Zhang W, et al. Exploration of essential gene functions via titratable promoter alleles. Cell 2004; 118: 31–44.

Schirwis E, Agbulut O, Vadrot N, Mouisel E, Hourdé C, Bonnieu A, et al. The beneficial effect of myostatin deficiency on maximal muscle force and power is attenuated with age. Exp. Gerontol. 2013; 48: 183–190.

Sharp NJ, Kornegay JN, Bartlett RJ, Hung WY, Dykstra MJ. Notexin-induced muscle injury in the dog. J. Neurol. Sci. 1993; 116: 73–81.

Vial G, Chauvin M-A, Bendridi N, Durand A, Meugnier E, Madec A-M, et al. Imeglimin Normalizes Glucose Tolerance and Insulin Sensitivity and Improves Mitochondrial Function in Liver of a High-Fat High-Sucrose Diet Mice Model. Diabetes 2014

Wilson LA, Cooper BJ, Dux L, Dubowitz V, Sewry CA. Expression of utrophin (dystrophin-related protein) during regeneration and maturation of skeletal muscle in canine X-linked muscular dystrophy. Neuropathol. Appl. Neurobiol. 1994; 20: 359–367.

## Legends to Supplementary Figures

**Supplementary Fig. 1.** Generation and analysis of *Hacd1* knockout mice. (**A**) Representation of the *Hacd1* locus, the structure of the targeting vector, and the organization of the rearranged *Hacd1* knockout allele after CRE-mediated recombination. (**B**) PCR genotyping of wild type, *Hacd1*$^{+/-}$ and *Hacd1*$^{-/-}$ mice. (**C**) RT-qPCR experiments for expression of *Hacd1* isoforms and *Hacd2* and *Hacd3* genes in TA muscles from wild type and *Hacd1*$^{-/-}$ mice (n = 3 for each condition). Note that expression of *Hacd2* and *Hacd3* genes was not modified in *Hacd1*-KO mice. *Hacd4* expression was not observed in either condition (data not shown). (**D**) Tibial length in wild type or *Hacd1*$^{+/-}$ (control) and *Hacd1*$^{-/-}$ 6 mo-old mice (n = 9 and 4 mice, respectively). (**E**) Mean specific maximal force (*i.e.,* the ratio of absolute force to muscle mass) of TA muscles from *Hacd1*$^{-/-}$, *Hacd1*$^{+/-}$ and wild type mice (n = 8, 6 and 8, respectively). (**F**) Photographs of a control and a *Hacd1*$^{-/-}$ 12-mo-old mouse. Note the kyphosis (red arrow) in the *Hacd1*$^{-/-}$ mouse. (**G**) H&E-stained transverse-sections of TA muscles at Day 6 after notexin injection in control and *Hacd1*$^{-/-}$ mice. Bars: 100 µm. Error bars correspond to standard error of the mean. *: $P < 0.05$; **: $P < 0.01$; ***: $P < 0.001$.

**Supplementary Fig. 2.** Weight gain and regeneration experiment in *HACD1*$^{cnm/cnm}$ dogs. (**A**) Total weight of *HACD1*$^{+/cnm}$ (control) and *HACD1*$^{cnm/cnm}$ pups of a same litter from birth to 28 days (n = 3 for each group). (**B**) Total weight of *HACD1*$^{+/cnm}$ (control) and *HACD1*$^{cnm/cnm}$ 2-mo-old pups (n = 7 and 13, respectively). Error bars correspond to standard error of the mean. *: $P < 0.05$. (**C**) H&E-staining on muscle sections before (Day 0) and at Day 4, 15, 30 or 90 after notexin injection in *HACD1*$^{+/cnm}$ (control) and *HACD1*$^{cnm/cnm}$ muscles (n=1 for each condition). Bars: 200 µm.

**Supplementary Fig. 3.** Progenitors in mice and expression of *Hacd* genes in myoblasts. (**A** and **B**) Immunofluorescence for PAX7 (in red) and Dystrophin (in green) on TA muscle cross-sections in control (**A**) and *Hacd1*$^{-/-}$ (**B**) mice. Nuclei are stained in blue. Asterisks point to PAX7-positive nuclei. (**C**) TA muscle area per PAX7-positive, satellite cell as observed in (**A** and **B**) (n = 3 mice per condition; more than 70 PAX7-positive cells examined per mouse). (**D**) RT-qPCR experiments for *Pax7* expression in TA muscles from control and *Hacd1*$^{-/-}$ mice (n = 4 for each condition). Error bars correspond to standard error of the mean. (**E**) Expression of *Hacd1* isoforms and *Hacd2* and *Hacd3* genes during C2C12 differentiation. Results of RT-qPCR experiments are expressed as a percentage of the level of the *Hacd1-fl* isoform at Day 2 (n = 3 for each condition). Error bars correspond to standard error of the mean. *: $P < 0.05$; **: $P < 0.01$; ***: $P < 0.001$.

**Supplementary Fig. 4.** Conformation, expression, HACD activity and interactions of HACD1 isoforms. (**A**) Topological structure of HACD1 isoforms predicted by the SOSUI program. Blue and pink residues are important and essential residues for HACD activity, respectively. Y171 residue (red square) was replaced by an A residue in HACD1-FL-Y171A construct. (**B**) Expression of Myc-tagged HACD1 isoforms in the ER of C2C12 myoblasts decorated with an anti-Calnexin antibody. Nuclei are stained in blue. (**C**) In mouse muscle section, a V5-tagged HACD1-FL isoform also localizes at the sarcoplasmic reticulum decorated with an anti-RyR1 antibody. Bars: 10 µm. (**D**) Co-Affinity Precipitation (Co-AP) experiments between HACD1 isoforms and other members of the Very Long Chain Fatty Acid elongation complex. GST-tagged HACD1 isoforms or the empty GST vector (baits) were co-expressed in HEK293T cells with 3xFLAG-tagged protein members of the complex, or with the MYC-tagged EIF2B1 taken as a negative control (preys). Eluates were revealed for GST (upper panel) and FLAG/MYC (mid panel). Expression of FLAG/MYC-tagged proteins was checked in total lysates (lower panel). Data are representative of two to three independent experiments. (**E**) Quantity of the 2,3-trans hexadecenoic acid produced by each HACD1 isoform (Fig. 6A) and expressed as a percentage of the initial quantity of substrate. Error bars correspond to standard error of the mean. (**F**) Quantities of 3xFLAG-tagged HACD1 isoforms in total yeast lysates (Fig. 6B) checked by western blotting (upper panel) and normalized to Pgk1 (lower panel).

**Supplementary Fig. 5.** Characterization of sh-*Hacd1* cells. (**A**) Expression of each *Hacd1* isoform by RT-qPCR in sh-*Hacd1* cells compared to control cells, both in proliferation and at Day 3 of differentiation. Data are expressed as a percentage of expression in control cells (n = 3 for each condition). Error bars correspond to standard error of the mean. *: $P < 0.05$; **: $P < 0.01$; ***: $P < 0.001$. (**B-C**) RT-qPCR experiments for *Myogenin* (**B**) and *Myosin, heavy polypeptide 2, skeletal muscle, adult (Myh2)* (**C**) gene expression in control and sh-*Hacd1* cells during differentiation. Significant differences in expression between proliferation and during differentiation are shown for each condition. (**D**) Immunofluorescence in sh-*Hacd1* cells at Day 5 of differentiation. Myosin heavy chains (MHC) are stained in green, actin in red and nuclei in blue. (**E**) Expression of Myc-tagged HACD1 isoforms in the ER of sh-*Hacd1* myoblasts decorated with an anti-Calnexin antibody. Nuclei are stained in blue; bars: 10 µm. (**F**) Total cell lysates from sh-*Hacd1* and isoform-expressing sh-*Hacd1* cells were subjected to immunoblotting using antibodies raised against Myc (upper panel) and Tubulin as a control of loading (lower panel).

**Supplementary Fig. 6.** Unsaturation level of phospholipid fatty acid and membrane fluidity in sh *Hacd1* cells. (**A**) Proportions of saturated (SFA), monounsaturated (MUFA) or polyunsaturated

(PUFA) fatty acids in proliferation (n = 3 for each condition; error bars correspond to standard error of the mean). (**B**) Fluorescence anisotropy (*r*, inverse of fluidity) measured at Day 3 of differentiation from 5°C to 37°C. For each cell type, the measured anisotropy increased when the temperature decreased, demonstrating that the DPH probe was in a lipid environment (n = 3 for each condition). Error bars correspond to standard error of the mean. *: $P < 0.05$; ***: $P < 0.001$.

## Supplementary Table 1

| Primer name * | Sequence |
|---|---|
| cHacd1-ex1F | 5'-CCTTCTACATCGCCATGACC-3' |
| cHacd1-ex7R | 5'-CACCTTGTTCAGGGGGATCATT-3' |
| cHacd1-fl-For | 5'-TATGGTGTGGCTCATCACACAT-3' |
| cHacd1-fl-Rev | 5'-TGGCCCATTTAATGAAGTATGGC-3' |
| cHacd1-d5-For | 5'-TATGGTGTGGCTCATCACACAT-3' |
| cHacd1-d5-Rev | 5'-CAATGGTATATATGAGGCCATGG-3' |
| cHacd1-167-For | 5'-TCGCCATGACCTCGGGATAC-3' |
| cHacd1-167-Rev | 5'-CAATGGTATATATGAGGCCATGG-3' |
| Hacd1-ex1F | 5'-GCCTGGCTCACCTTCTACAAT-3' |
| Hacd1-ex7R | 5'-CGTGGAGCACCTTTCTTCTC-3' |
| Hacd1-For | 5'-ATATCCCGTTGGAGTTGCTG-3' |
| Hacd1-Rev | 5'-CGTGGAGCACCTTTCTTCTC-3' |
| Hacd1-fl-For | 5'-ATGAAGAGAGCGTGGTGCTT-3' |
| Hacd1-fl-Rev | 5'-AAGGCGGCGTATATTGTGAG-3' |
| Hacd1-For-2 | 5'-ATGGCGTCCAGTGAGGAGGACGGC-3' |
| Hacd1-Rev-2 | 5'-TTAATCGTCCTTCTCCGCGATC-3' |
| Hacd1-d5-For | 5'-TCACAGTATAAAACCCATACAATT-3' |
| Hacd1-d5-Rev | 5'-GAGGCCATGGTTATGAGAAG-3' |
| Hacd1-167-For | 5'-AATATCGCCATGACGGCCGGATAC-3' |
| Hacd1-167-Rev | 5'-CAACGGTATATAGGAGGCCATG-3' |
| cycloB-For (*Ppib* gene) | 5'-GATGGCACAGGAGGAAAGAG-3' |
| cycloB-Rev (*Ppib* gene) | 5'-AACTTTGCCGAAAACCACAT-3' |
| Hacd2-For | 5'-TGCTATAGGGATTGTGCCATC-3' |
| Hacd2-rev | 5'-ACGGATAATTTCCGTGATTGTCC-3' |
| Hacd3-For | 5'-GACGTGCAGAACCCTGCTATC-3' |
| Hacd3-Rev | 5'-CTTCTGGACTGTGATGTTCACC-3' |
| Hacd4-For | 5'-CAGCTCACAGAGAGAGTGATC-3' |
| Hacd4-Rev | 5'-GAGTGTTTGACTGAGCCATGTC-3' |
| Myogenin-For | 5'-CTACAGGCCTTGCTCAGCTC-3' |
| Myogenin-Rev | 5'-AGATTGTGGGCGTCTGTAG-3' |
| Myh2-For | 5'-ACTGGAGGGTGAGGTAGAGAG-3' |
| Myh2-Rev | 5'-GCCTCCTCAGCTTGTCTCTTG-3' |
| Gapdh-For | 5'-TGAAGGTCGGTGTCAACGGATTTGGC-3' |
| Gapdh-Rev | 5'-CATGTAGGCCATGAGGTCCACCAC-3' |
| Pax7-For | 5'-AGGCCTTCGAGAGGACCCAC-3' |
| Pax7-Rev | 5'-CTGAACCAGACCTGGACGCG-3' |
| TBP-For | 5'-ATCCCAAGCGATTTGCTG-3' |
| TBP-Rev | 5'-CCTGTGCACACCATTTTTCC-3' |
| Hprt1-For | 5'-GTTAAGCAGTACAGCCCCAAA-3' |
| Hprt1-Rev | 5'-AGGGCATATCCAACAACAAACTT-3' |

*c: canine sequence; otherwise: mouse sequence

## Supplementary Table 2

A

| Phospholipids (%) | Proliferation: Control (a) | Proliferation: *sh-Hacd1* (b) | Proliferation: *sh-Hacd1+fl* (c) | Proliferation: *sh-Hacd1+d5* (d) | Differentiation (Day 3): Control (e) | Differentiation (Day 3): *sh-Hacd1* (f) | Differentiation (Day 3): *sh-Hacd1+fl* (g) |
|---|---|---|---|---|---|---|---|
| PC | 55.067±0.213$^{b,d,f,g}$ | 53.263±0.219$^{a,d,f,g}$ | 53.650±0.411$^{d,f,g}$ | 46.935±0.076$^{g}$ | 48.368±2.175 | 47.328±1.214$^{a,b,c}$ | 50.391±0.064$^{a,b,c,d}$ |
| PI | 20.129±0.381$^{c,d,e,g}$ | 17.967±0.942$^{e,g}$ | 14.512±1.403$^{a,e,f,g}$ | 15.484±0.988$^{a,e,f,g}$ | 30.913±2.386$^{a,b,c,d}$ | 22.315±1.736$^{c,d}$ | 24.834±0.156$^{a,b,c,d}$ |
| PE | 9.108±0.368$^{b,c,d,g}$ | 11.905±0.328$^{a,e}$ | 12.322±0.551$^{a,e}$ | 12.048±0.251$^{a,e}$ | 8.510±0.365$^{b,c,d,g}$ | 11.334±1.040 | 11.499±0.180$^{a,e}$ |
| PS | 3.202±0.046$^{g}$ | 3.060±0.026$^{g}$ | 2.823±0.159$^{g}$ | 2.920±0.177$^{g}$ | 3.244±0.100$^{g}$ | 3.557±0.343 | 3.840±0.103$^{a,b,c,d,e}$ |
| SM | 5.619±0.144$^{b,c,d,f,g}$ | 7.671±0.179$^{a,d,e,f}$ | 9.304±0.928$^{a,d,e}$ | 14.500±0.960$^{a,b,c,e,f,g}$ | 5.416±0.114$^{b,c,d,f,g}$ | 9.111±0.217$^{a,b,d,e,g}$ | 7.093±0.024$^{a,d,e,f}$ |
| LPC | 4.718±0.016$^{e,g}$ | 4.080±0.221$^{d,e,g}$ | 4.924±0.069$^{e,g}$ | 5.648±0.201$^{b,e,g}$ | 2.246±0.132$^{a,b,c,d,f,g}$ | 5.066±0.559$^{e,g}$ | 1.572±0.047$^{a,b,c,d,e,f}$ |
| LPE | 2.156±0.145$^{e,f,g}$ | 2.053±0.141$^{e,f,g}$ | 2.464±0.019$^{e,f,g}$ | 2.463±0.232$^{e,f,g}$ | 1.304±0.046$^{a,b,c,d,g}$ | 1.289±0.086$^{a,b,c,d,g}$ | 0.772±0.011$^{a,b,c,d,e,f}$ |

B

| LPC (% of phospholipids) | Proliferation: Control (a) | Proliferation: *sh-Hacd1* (b) | Proliferation: *sh-Hacd1+fl* (c) | Proliferation: *sh-Hacd1+d5* (d) | Differentiation (Day 3): Control (e) | Differentiation (Day 3): *sh-Hacd1* (f) | Differentiation (Day 3): *sh-Hacd1+fl* (g) |
|---|---|---|---|---|---|---|---|
| 14:0 | 0.104±0.001$^{c,g}$ | 0.092±0.005$^{c,d,g}$ | 0.115±0.003$^{a,b,e,g}$ | 0.169±0.017$^{b,e,g}$ | 0.088±0.004$^{c,d,g}$ | 0.562±0.116 | 0.071±0.003$^{a,b,c,d,e}$ |
| 16:0 | 1.799±0.007$^{d,e,g}$ | 1.610±0.098$^{d,e,g}$ | 1.937±0.051$^{d,e,g}$ | 2.174±0.028$^{a,b,c,e,g}$ | 0.953±0.058$^{a,b,c,d,f,g}$ | 2.518±0.279$^{e,g}$ | 0.648±0.020$^{a,b,c,d,e,f}$ |
| 18:0 | 0.518±0.003$^{e,f,g}$ | 0.439±0.025$^{d,e,f,g}$ | 0.513±0.008$^{e,f,g}$ | 0.663±0.061$^{b,e,g}$ | 0.313±0.018$^{a,b,c,d,f,g}$ | 1.275±0.178$^{a,b,c,e,g}$ | 0.236±0.011$^{a,b,c,d,e,f}$ |
| 18:1 | 2.064±0.022$^{b,e,f,g}$ | 1.722±0.083$^{a,c,e,f,g}$ | 2.099±0.039$^{b,e,f,g}$ | 2.068±0.139$^{e,f,g}$ | 0.770±0.004$^{a,b,c,d,g}$ | 0.588±0.087$^{a,b,c,d}$ | 0.491±0.012$^{a,b,c,d,e}$ |
| 18:2 | 0.149±0.019 | 0.150±0.006$^{d,e,g}$ | 0.153±0.017 | 0.218±0.000$^{b,e,g}$ | 0.099±0.009$^{b,d}$ | 0.197±0.031 | 0.109±0.003$^{a,b,c,d,e}$ |
| 20:4 | 0.082±0.003$^{d,e,f,g}$ | 0.094±0.003$^{d,e,g}$ | 0.102±0.007$^{d,e,g}$ | 0.250±0.003$^{a,b,c,e,f,g}$ | 0.009±0.013$^{a,b,c,d,f}$ | 0.111±0.005$^{a,d,e,g}$ | 0.014±0.001$^{a,b,c,d,e,f}$ |

**Legend.** Repartition of (**A**) phospholipid species and (**B**) LPC species in control, *sh-Hacd1* and isoform expressing sh-*Hacd1* cells in proliferation and at Day 3 of differentiation. The amount of each species is expressed as its mole fraction to the total content (mol %; n = 3 for each condition). Superscript letters indicate significant differences ($P < 0.05$) with values reported in columns named using a one-letter-code. For example, the value of LPC in differentiating control cells (2.246±0.132$^{a,b,c,d,f,g}$) is significantly different from all the other values [(a,b,c,d,f,g) columns], and LPC in differentiating *sh-Hacd1* is significantly different from values of LPC in control and *sh-Hacd1+fl* cells [(e,g) columns].

# Supplementary Table 3

A

| Fatty acids (%) | Proliferation | | | | Differentiation (Day 3) | | |
|---|---|---|---|---|---|---|---|
| | Control (a) | *sh-Hacd1* (b) | *sh-Hacd1+fl* (c) | *sh-Hacd1+d5* (d) | Control (e) | *sh-Hacd1* (f) | *sh-Hacd1+fl* (g) |
| 10:0 | **0.170±0.029$^{e}$** | 0.170±0.04 | **0.087±0.009$^{d}$** | **0.249±0.045$^{c}$** | **0.069±0.011$^{a,g}$** | 0.438±0.142 | **0.334±0.039$^{e}$** |
| 12:0 | **0.357±0.042$^{c,d}$** | **0.412±0.051$^{c,d}$** | **0.163±0.003$^{a,b,d}$** | **0.607±0.02$^{a,b,c}$** | **0.251±0.067$^{f,g}$** | **1.149±0.184$^{e}$** | **0.694±0.106$^{e}$** |
| 14:0 | **3.371±0.294$^{e}$** | **3.338±0.134$^{c}$** | **2.914±0.072$^{b}$** | 3.216±0.153 | **6.308±0.172$^{a,f,g}$** | **4.977±0.263$^{e}$** | **4.741±0.182$^{e}$** |
| 16:0 | **34.701±1.509$^{c,d,e}$** | **37.257±0.339$^{c,d}$** | **27.492±0.497$^{a,b,d}$** | **42.282±0.821$^{a,b,c}$** | **23.939±1.009$^{a,f,g}$** | **40.131±0.072$^{e,g}$** | **33.915±0.904$^{e,f}$** |
| 18:0 | **16.185±0.295$^{b,c,e}$** | **14.765±0.287$^{a,c}$** | **11.695±0.598$^{a,b,d}$** | **16.063±0.801$^{c}$** | **26.232±2.050$^{a,f,g}$** | **18.031±0.234$^{e,g}$** | **11.028±0.642$^{e,f}$** |
| 20:0 | **0.066±0.004$^{b,d,e}$** | **0.085±0.004$^{a,c}$** | **0.066±0.002$^{b,d}$** | **0.114±0.012$^{a,c}$** | **0.209±0.013$^{a,g}$** | **0.224±0.009$^{g}$** | **0.120±0.020$^{e,f}$** |
| 22:0 | **0.030±0.001$^{b,c,d,e}$** | **0.042±0.001$^{a,c}$** | **0.035±0.000$^{a,b,d}$** | **0.060±0.007$^{a,c}$** | **0.025±0.001$^{a,f,g}$** | **0.104±0.009$^{e,g}$** | **0.058±0.010$^{e,f}$** |
| 24:0 | **0.040±0.002$^{b,c,d,e}$** | **0.070±0.003$^{a,d}$** | **0.075±0.001$^{a}$** | **0.115±0.015$^{a,b}$** | **0.010±0.001$^{a,f,g}$** | **0.159±0.015$^{e,g}$** | **0.094±0.015$^{e,f}$** |
| 26:0 | **0.009±0.002$^{b,d,e}$** | **0.017±0.001$^{a,c}$** | **0.009±0.000$^{b,d}$** | **0.028±0.005$^{a,c}$** | **0.001±0.000$^{a,g}$** | 0.037±0.019 | **0.026±0.004$^{e}$** |
| 16:1 n-7 | **4.136±0.748$^{c,d,e}$** | **3.891±0.146$^{c,d}$** | **10.196±0.298$^{a,b,d}$** | **2.201±0.087$^{a,b,c}$** | **8.574±0.583$^{a,f}$** | **3.882±0.167$^{e,g}$** | **7.063±0.595$^{f}$** |
| 16:1 n-9 | **2.769±0.2$^{c}$** | **3.135±0.097$^{c}$** | **3.919±0.122$^{a,b,d}$** | **2.892±0.266$^{c}$** | 3.283±0.441 | 4.836±0.538 | 4.775±0.325 |
| 18:1 n-7 | **4.650±0.374$^{c,d,e}$** | **4.137±0.214$^{c,d}$** | **8.099±0.256$^{a,b,d}$** | **2.921±0.141$^{a,b,c}$** | **7.684±0.582$^{a,f,g}$** | **2.255±0.102$^{e,g}$** | **4.637±0.464$^{e,f}$** |
| 18:1 n-9 | **19.364±1.097$^{c,d}$** | **19.636±0.469$^{c,d}$** | **26.164±0.308$^{a,b,d}$** | **15.196±0.439$^{a,b,c}$** | **16.06±0.641$^{g}$** | **15.131±0.769$^{g}$** | **22.226±1.081$^{e,f}$** |
| 20:1 | **0.180±0.016$^{b,c,d}$** | **0.309±0.037$^{a}$** | 0.238±0.020 | **0.344±0.039$^{a}$** | **0.186±0.027$^{f}$** | **0.710±0.106$^{e}$** | 0.481±0.109 |
| 22:1 | **0.035±0.001$^{b,d,e}$** | **0.055±0.004$^{a,c}$** | **0.039±0.002$^{b,d}$** | **0.065±0.007$^{a,c}$** | **0.065±0.008$^{a,f}$** | **0.118±0.016$^{e}$** | 0.084±0.022 |
| 24:1 | **0.011±0.001$^{b,c,d}$** | **0.019±0.001$^{a,c,d}$** | **0.022±0.001$^{a,b,d}$** | **0.031±0.002$^{a,b,c}$** | 0.016±0.002 | 0.021±0.001 | 0.020±0.003 |
| 18:2 | **1.22±0.05$^{b,d,e}$** | **1.435±0.007$^{a,c}$** | **1.358±0.016$^{b}$** | **1.469±0.056$^{a}$** | **2.427±0.266$^{a,f,g}$** | **3.400±0.193$^{e}$** | **3.879±0.193$^{e}$** |
| 18:3 | 0.143±0.021 | **0.133±0.001$^{d}$** | **0.140±0.003$^{d}$** | **0.100±0.004$^{b,c}$** | **0.156±0.023$^{f,g}$** | **0.283±0.020$^{e,g}$** | **1.028±0.123$^{e,f}$** |
| 20:3 | **2.367±0.086$^{c,d,e}$** | **2.225±0.057$^{c}$** | **2.759±0.091$^{a,b,d}$** | **1.997±0.081$^{a,b,c}$** | **0.653±0.081$^{a,f,g}$** | **1.251±0.053$^{e,g}$** | **2.142±0.142$^{e,f}$** |
| 20:4 | **4.6±0.479$^{c,e}$** | **4.087±0.074$^{c,d}$** | **2.119±0.060$^{a,b,d}$** | **5.370±0.177$^{b,c}$** | **1.677±0.287$^{a}$** | 1.152±0.048 | 1.185±0.073 |
| 20:5 | **0.442±0.01$^{c,d,e}$** | **0.438±0.006$^{c,d}$** | **0.372±0.013$^{a,b,d}$** | **0.497±0.012$^{a,b,c}$** | **0.997±0.191$^{a}$** | 0.715±0.034 | 1.059±0.068 |
| 22:4 | **0.533±0.087$^{c,e}$** | **0.454±0.006$^{c,d}$** | **0.143±0.002$^{a,b,d}$** | **0.540±0.002$^{b,c}$** | **0.082±0.013$^{a}$** | 0.114±0.006 | 0.088±0.010 |
| 22:5 | **2.491±0.243$^{c,e}$** | **2.060±0.024$^{c,d}$** | **0.959±0.008$^{a,b,d}$** | **2.167±0.015$^{b,c}$** | **0.578±0.109$^{a}$** | 0.530±0.025 | 0.532±0.041 |
| 22:6 | **2.104±0.092$^{b,c,d,e}$** | **1.800±0.039$^{a,c,d}$** | **0.925±0.033$^{a,b,d}$** | **1.441±0.051$^{a,b,c}$** | **0.484±0.082$^{a}$** | **0.344±0.013$^{g}$** | **0.500±0.048$^{f}$** |
| 24:4 | ND | ND | ND | ND | 0.007±0.000 | ND | ND |
| 24:5 | ND | ND | ND | ND | 0.005±0.001 | ND | ND |
| 24:6 | **0.026±0.003$^{c,e}$** | **0.029±0.001$^{c,d}$** | **0.014±0.000$^{a,b,d}$** | **0.025±0.000$^{b,c}$** | **0.008±0.001$^{a}$** | 0.006±0.000 | 0.007±0.001 |

B

| Fatty acids (%) | Proliferation | | | | Differentiation (Day 3) | | |
|---|---|---|---|---|---|---|---|
| | Control (a) | *sh-Hacd1* (b) | *sh-Hacd1+fl* (c) | *sh-Hacd1+d5* (d) | Control (e) | *sh-Hacd1* (f) | *sh-Hacd1+fl* (g) |
| C18-C26/C10-C16 | **1.198±0.026$^{b,d,e,f,g}$** | **1.074±0.021$^{a,c,d,e,f,g}$** | **1.185±0.016$^{b,d,e,f,g}$** | **0.926±0.037$^{a,b,c,e,f}$** | **1.357±0.014$^{a,b,c,d,f,g}$** | **0.805±0.035$^{a,b,c,d,e,g}$** | **0.921±0.028$^{a,b,c,e,f}$** |

C

| Fatty acids (%) | Proliferation | | | | Differentiation (Day 3) | | |
|---|---|---|---|---|---|---|---|
| | Control (a) | *sh-Hacd1* (b) | *sh-Hacd1+fl* (c) | *sh-Hacd1+d5* (d) | Control (e) | *sh-Hacd1* (f) | *sh-Hacd1+fl* (g) |
| SFA | **54.928±1.670$^{c,d,f}$** | **56.157±0.815$^{c,d,f}$** | **42.536±0.685$^{a,b,d,e,f,g}$** | **62.734±0.878$^{a,b,c,g}$** | **57.048±3.265$^{c}$** | **65.252±1.935$^{a,b,c,g}$** | **51.010±1.774$^{c,d,f}$** |
| MUFA | **31.146±2.387$^{c,d}$** | **31.181±0.662$^{c,d,g}$** | **48.677±0.511$^{a,b,d,e,f,g}$** | **23.651±0.581$^{a,b,c,e,g}$** | **35.872±2.283$^{c,d,f}$** | **26.953±1.563$^{c,e,g}$** | **39.286±1.699$^{b,c,d,f}$** |
| PUFA | **13.926±0.757$^{c,e,f,g}$** | **12.662±0.175$^{c,e,f,g}$** | **8.788±0.220$^{a,b,d,g}$** | **13.615±0.350$^{c,e,f,g}$** | **7.080±0.991$^{a,b,d}$** | **7.795±0.379$^{a,b,d,g}$** | **10.420±0.318$^{a,b,c,d,f}$** |

**Legend.** Phospholipid fatty acid sorted by their content (**A**), length ratio (**B**) and saturation classes (**C**) of control, *sh-Hacd1* and isoform expressing sh-*Hacd1* cells in proliferation and at Day 3 of differentiation. Total phospholipid fatty acid methyl esters are presented depending of their aliphatic chain and saturation degree. The amount of each fatty acid is expressed as its mole fraction to the total free fatty acid content (mol %; n = 3 for each condition). Superscript letters indicate significant differences ($P < 0.05$) with values reported in columns named using the one-letter-code described in Supplementary Table 2.

# Supplementary Figure 1

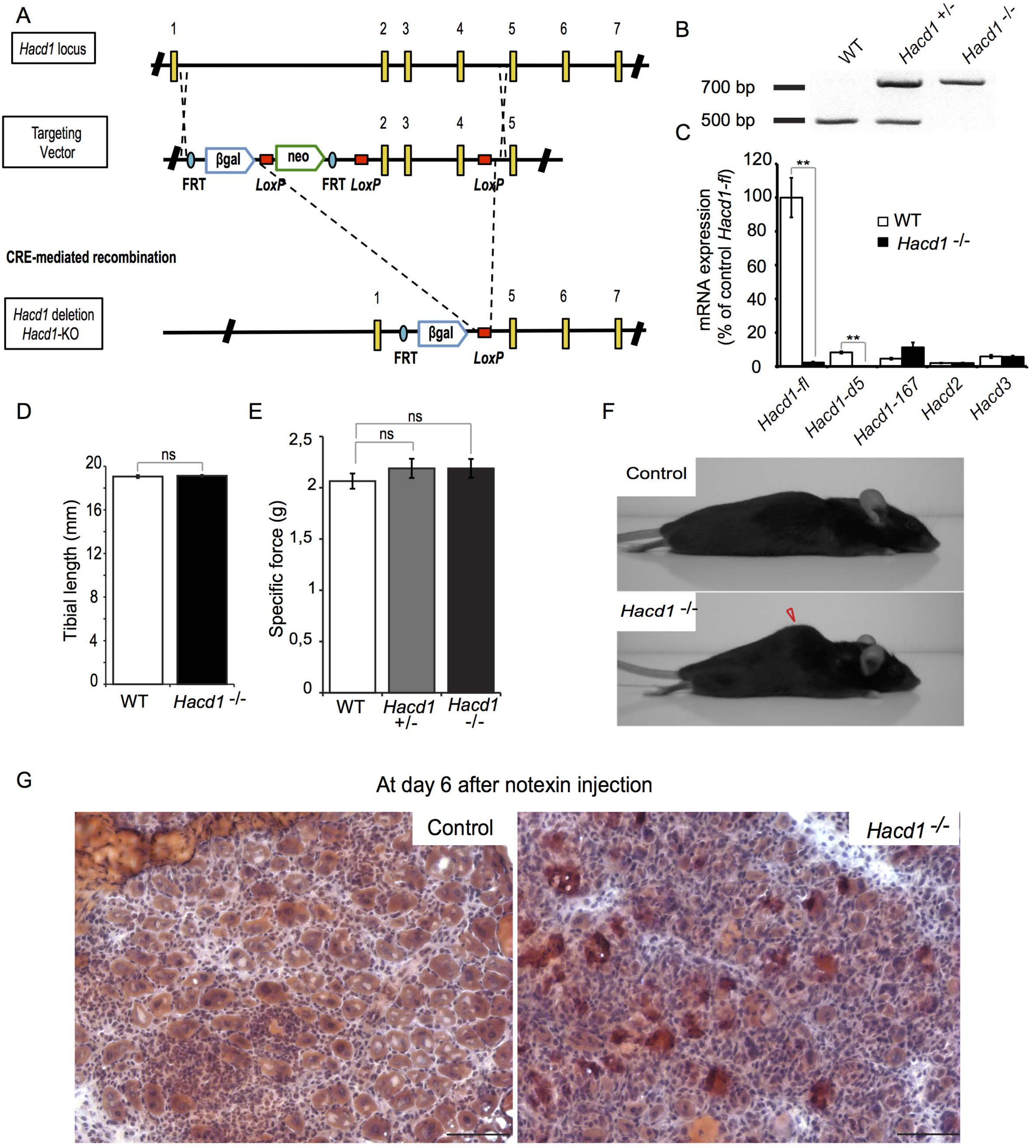

Supplementary Figure 2

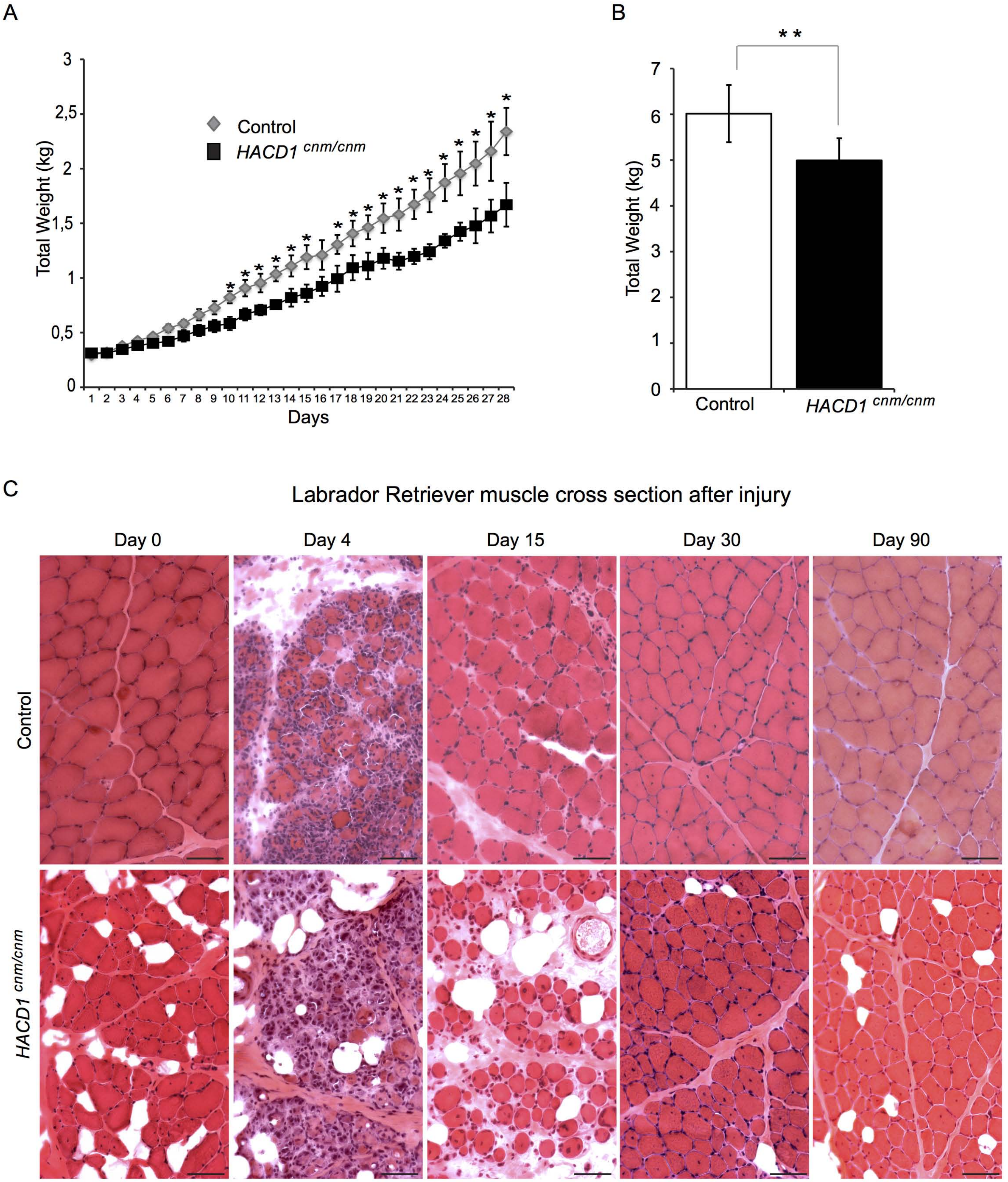

# Supplementary Figure 3

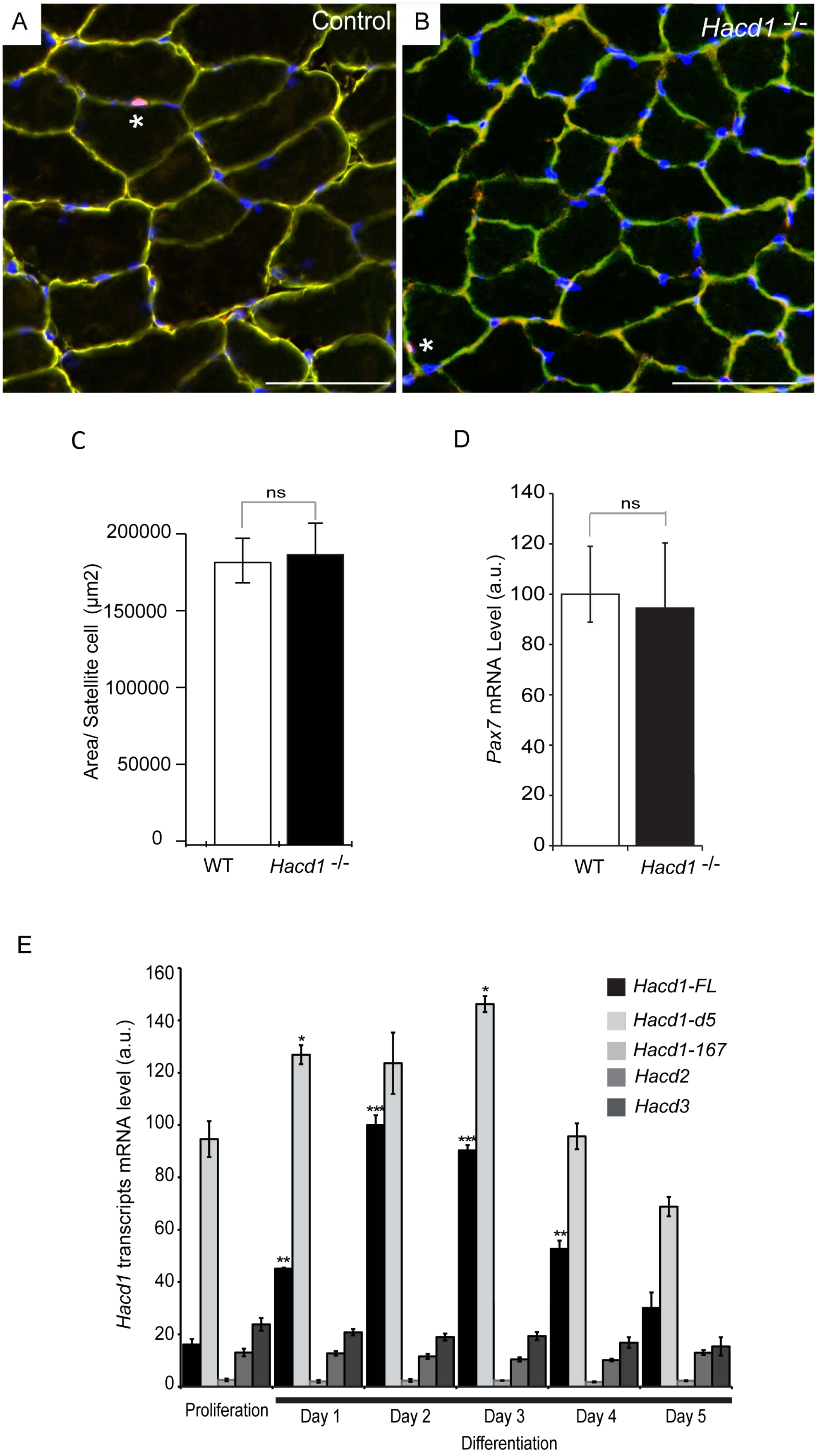

Supplementary Figure 4

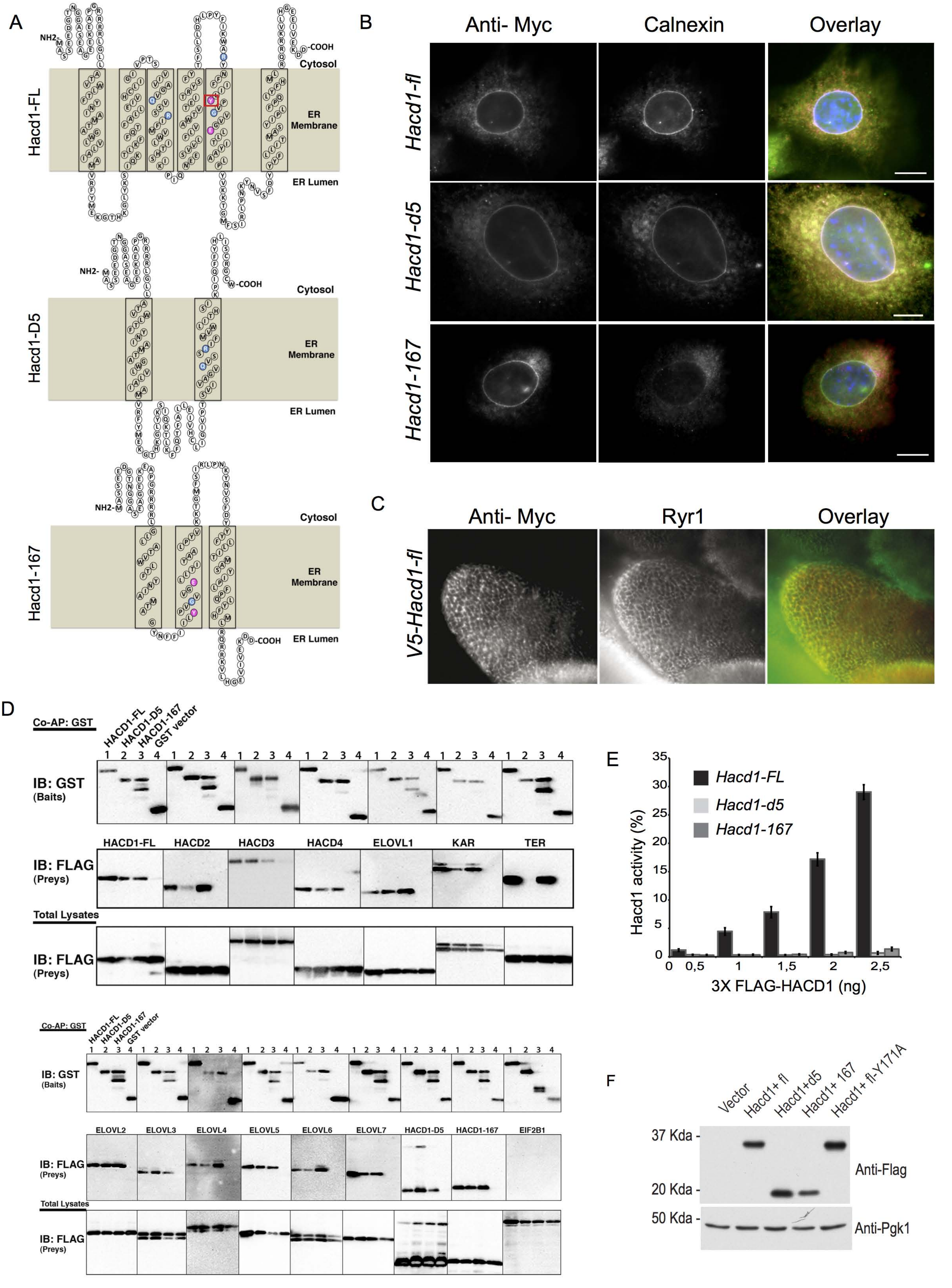

# Supplementary Figure 5

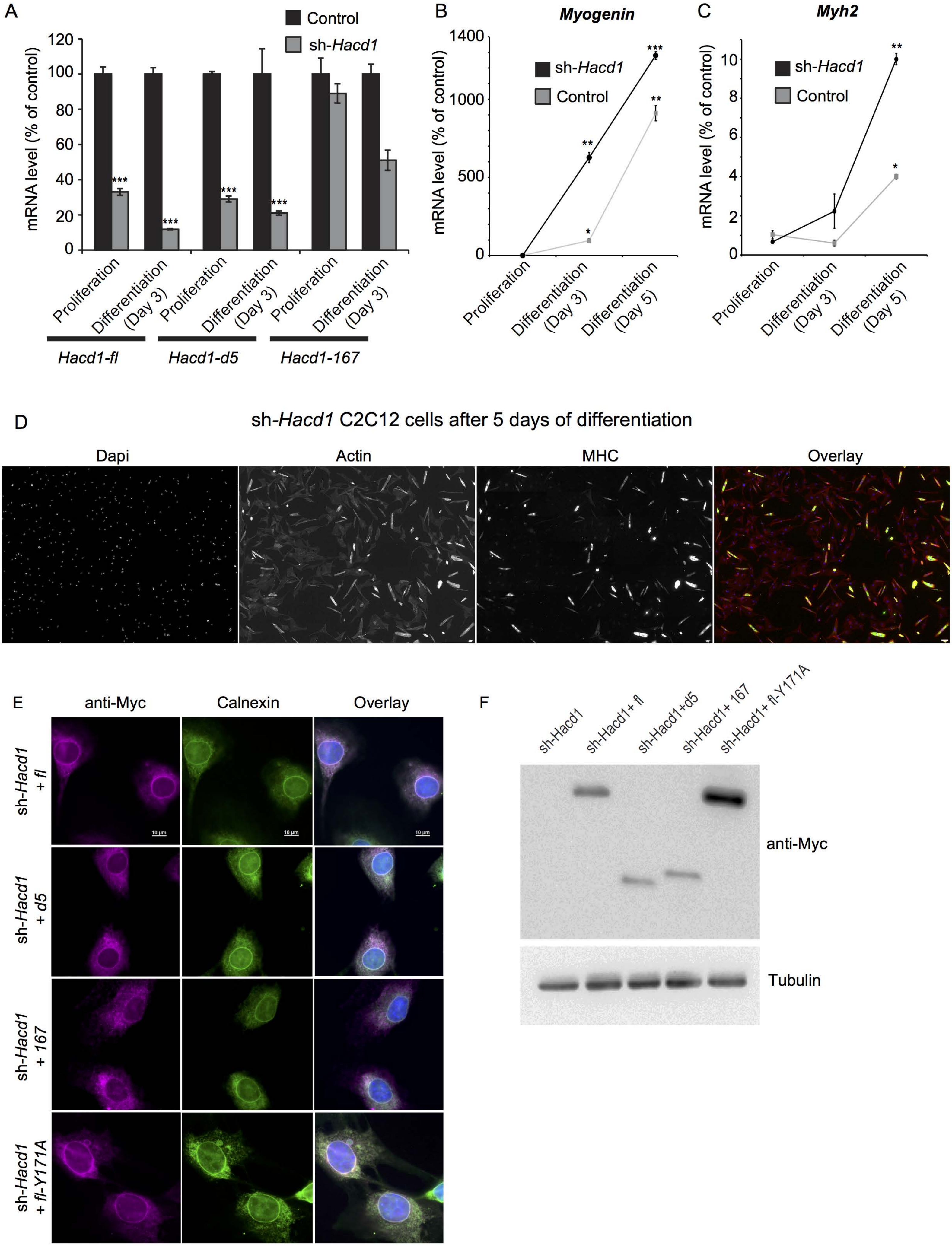

Supplementary Figure 6

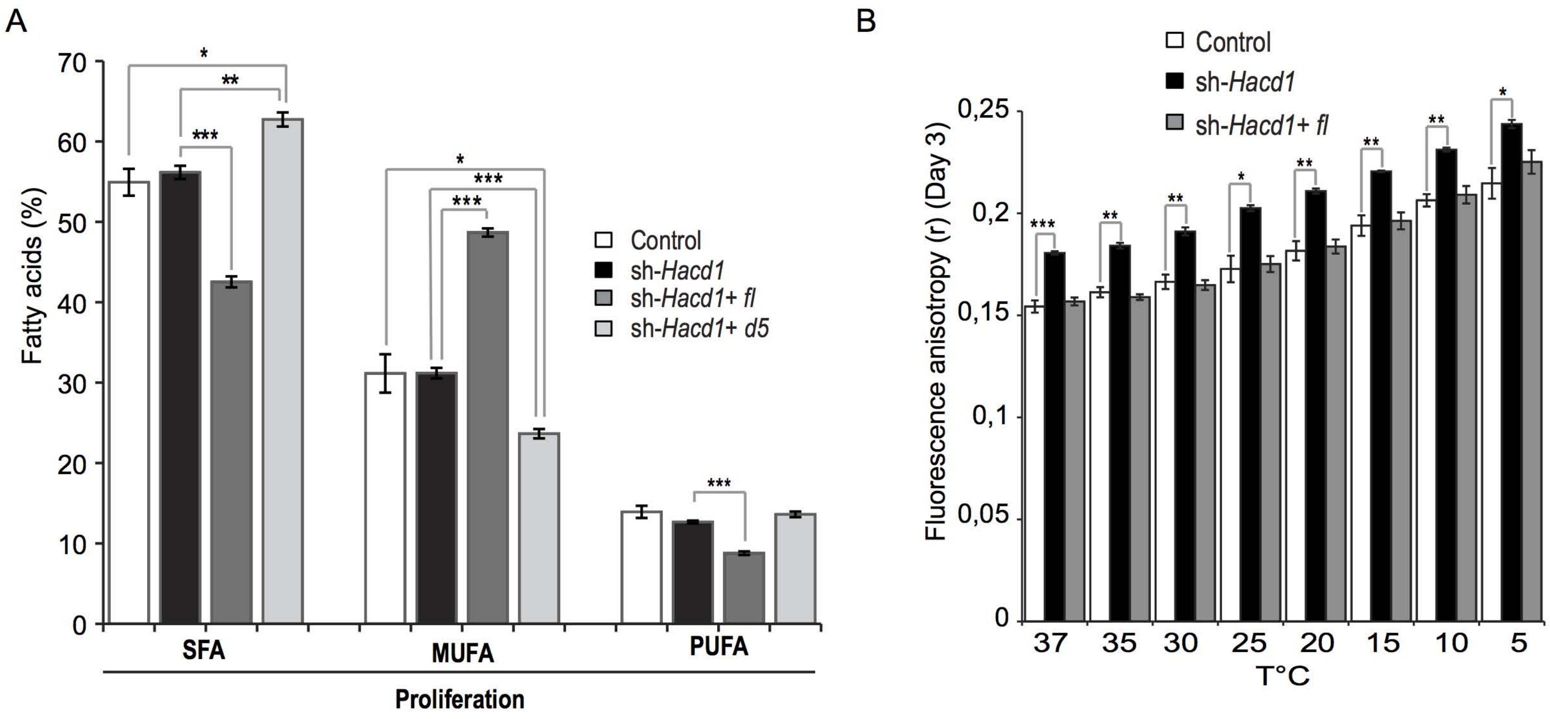

Supplement: Supplementary Data [file supp_mjv049_mjv049supp_data.pdf]
